# Trophoblast glycoprotein is a new candidate gene for Parkinson’s disease

**DOI:** 10.1038/s41531-021-00252-0

**Published:** 2021-12-07

**Authors:** Sanghyun Park, Jeong-Eun Yoo, Gyu-Bum Yeon, Jin Hee Kim, Jae Souk Lee, Sung Kyoung Choi, Young-Gi Hwang, Chan Wook Park, Myung Soo Cho, Jongwan Kim, Dokyun Na, Hyung Wook Kim, Dae-Sung Kim, Dong-Wook Kim

**Affiliations:** 1 Department of Physiology, Yonsei University College of Medicine, Seoul, South Korea; 2 Severance Biomedical Research Institute, Yonsei University College of Medicine, Seoul, South Korea; 3 Brain Korea 21 PLUS Program for Medical Science, Yonsei University College of Medicine, Seoul, South Korea; 4 Department of Biotechnology, College of Life Science and Biotechnology, Korea University, Seoul, South Korea; 5 S. Biomedics Co., Ltd, Seoul, South Korea; 6 Department of Biomedical Engineering, School of Integrative Engineering, Chung-Ang University, Seoul, South Korea; 7 Department of Bio-integrated Science and Technology, College of Life Sciences, Sejong University, Seoul, South Korea; 8 Department of Pediatrics, Korea University College of Medicine, Guro Hospital, Seoul, South Korea

**Keywords:** Parkinson's disease, Neurodegeneration

## Abstract

Parkinson’s disease (PD) is a movement disorder caused by progressive degeneration of the midbrain dopaminergic (mDA) neurons in the substantia nigra pars compacta (SNc). Despite intense research efforts over the past decades, the etiology of PD remains largely unknown. Here, we discovered the involvement of trophoblast glycoprotein (*Tpbg*) in the development of PD-like phenotypes in mice. *Tpbg* expression was detected in the ventral midbrain during embryonic development and in mDA neurons in adulthood. Genetic ablation of *Tpbg* resulted in mild degeneration of mDA neurons in aged mice (12–14 months) with behavioral deficits reminiscent of PD symptoms. Through in silico analysis, we predicted potential TPBG-interacting partners whose functions were relevant to PD pathogenesis; this result was substantiated by transcriptomic analysis of the SNc of aged *Tpbg* knockout mice. These findings suggest that *Tpbg* is a new candidate gene associated with PD and provide a new insight into PD pathogenesis.

## Introduction

Parkinson’s disease (PD) is the most common neurodegenerative movement disorder, affecting approximately 1% of the population aged above 65 years. It is clinically characterized by bradykinesia, resting tremor, rigidity, and postural instability^1,2^. The pathological hallmarks of PD include progressive degeneration of nigrostriatal dopaminergic neurons and the presence of cytoplasmic and neuritic deposits of α-synuclein (α-SYN)^3^. Accumulating evidence suggests that the pathological form of α-SYN might trigger neuroinflammation through microglial activation, contributing to the apoptotic death of dopaminergic neurons^4,5^. The combination of dopaminergic neuronal loss and α-SYN pathology is believed to lead to dopamine depletion in the striatum, resulting in the aforementioned motor symptoms^6^. Most PD occurrences are idiopathic; however, the identification of genetic factors that cause inheritable forms of PD has yielded crucial insights into possible pathogenic mechanisms. Many of the genetic factors have been proposed to be involved in mitochondrial dysfunction, increased oxidative stress, aberrant protein aggregation, and defective proteasome degradation^7,8^. Despite extensive research conducted in this area, only a few genes are known to be directly involved in monogenic forms of PD, and 90 genetic variants identified by meta-analysis of genome-wide association studies accounts for 16−36% of the risk of idiopathic PD depending on prevalence^9^.

Trophoblast glycoprotein (TPBG), also known as Wnt-activated inhibitory factor 1 (WAIF1), is a 72 kDa, heavily *N*-glycosylated, single-pass transmembrane protein^10–12^. It is highly expressed not only in trophoblast cells and carcinoma but also in normal adult tissues, including the ovary, bone, retina, and brain^13,14^. TPBG has been primarily studied in embryonic stem (ES) cell differentiation and cancer metastasis^15–18^. During embryonic development, TPBG acts as a feedback inhibitor of canonical Wnt signaling by interfering with the internalization of low-density lipoprotein receptor-related protein 6 (LRP6), a key component of the LRP5/LRP6/Frizzled co-receptor group, and enhances the activation of non-canonical Wnt signaling by stimulating the functions of Dickkopf-related protein 1 (DKK1)^19^. Additionally, TPBG modulates cell adhesion, cytoskeletal organization, and mobility by facilitating functional C-X-C chemokine receptor type 4 (CXCR4) expression, leading to C-X-C motif chemokine 12 (CXCL12)-mediated chemotaxis in differentiating ES cells, embryonic fibroblasts, and cancer cells^15–18,20^. More recently, analysis of a single-cell RNA sequencing (RNA-seq) dataset of the developing human ventral midbrain (VM) obtained from 6 to 11 week embryos revealed that *TPBG* is significantly expressed in early neural progenitor cells of the floor plate and in a subset of dopaminergic neurons (referred to as “DA2” population) whose fate is determined to substantia nigra pars compacta (SNc) and ventral tegmental area (VTA) 1 and 2 after birth^21^, implicating the role of *TPBG* in the development of midbrain dopaminergic (mDA) neurons. Furthermore, we have recently discovered that TPBG expression is specifically enriched in mDA precursors differentiated from human pluripotent stem cells (hPSCs) and proposed that it can be used as a marker for mDA precursor isolation from neural progenies of hPSCs^22^. Given its specific expression in the mDA population, *TPBG* has been considered as one of the PD-related genes^23,24^. Indeed, a study analyzing gene expression in postmortem tissues showed that *TPBG* was significantly downregulated in the SNc of patients with PD than in the SNc of age-matched healthy subjects^23^. Despite the evidence, detailed information including spatial and temporal expression of *TPBG* during embryonic development and its role in fate determination or maintenance of mDA neurons remain largely unknown; more importantly, the involvement of *TPBG* in PD etiology remains circumstantial.

In this study, we investigated the spatiotemporal expression of *Tpbg* in the VM of developing mouse embryos and adult mice. Our results demonstrate that *Tpbg* was specifically expressed in the VM region of developing embryos and in mDA neurons of the SNc and VTA in adults. In addition, we found that genetic ablation of *Tpbg* negatively affected the survival of mDA neurons in adult mice and resulted in motor impairments reminiscent of PD symptoms. This study provides evidence of the specific expression of *Tpbg* in developing and mature mDA neurons and its involvement in the maintenance of the mDA neuronal population, suggesting that *Tpbg* is a candidate gene associated with PD etiology.

## Results

### *Tpbg* is expressed in the developing mouse ventral midbrain

**Fig. 1:**
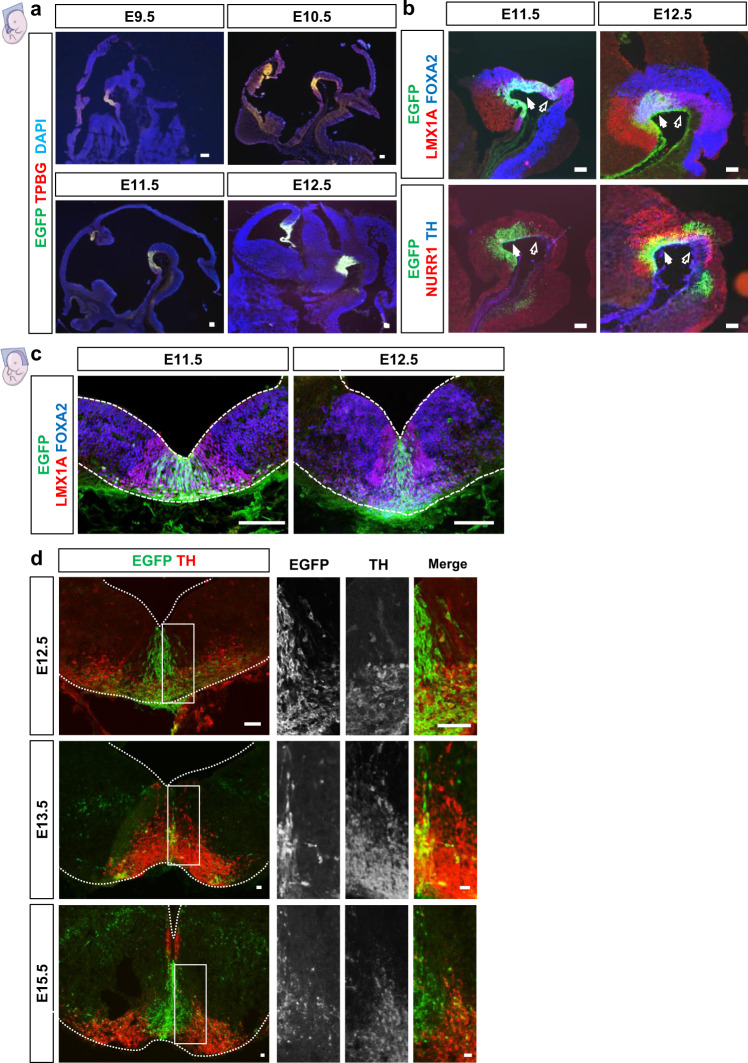
Analysis of spatiotemporal expression of TPBG during mDA development. **a** Between E9.5 and E12.5, immunohistochemical staining on *Tpbg*-EGFP mice embryo revealed that TPBG was expressed in the floor plate of the developing midbrain and co-expressed with EGFP in a sagittal manner (Scale bars: 100 μm). **b** At E11.5 and E12.5, immunohistochemical staining revealed that EGFP was co-expressed with LMX1A, FOXA2, NURR1, and TH in the ventral midbrain (VM). Solid arrows indicate the rostral part and open arrows indicate the caudal part of the VM region (Scale bars: 100 μm). **c** Immunohistochemical staining in the coronal section at E11.5 and E12.5 revealed that EGFP was expressed in the ventral midline, overlapping with FOXA2 and LMX1A expression (Scale bars: 100 μm). **d** Between E12.5 and E15.5, EGFP-expressing cells showed bipolar cell morphology with leading processes directed either radially or tangentially in the mDA domain (Scale bars: 50 μm). E embryonic day; mDA midbrain dopaminergic.

The spatiotemporal expression of *Tpbg* in the developing VM was investigated using immunohistochemical analysis of mouse brain tissue at different embryonic stages [embryonic day (E)9.5–E15.5] (Fig. 1). To pinpoint the location of *Tpbg* expression, a transgenic mouse strain in which enhanced green fluorescent protein (EGFP) is expressed under the *Tpbg* promoter was used. Immunohistochemical staining for TPBG showed that most EGFP^+^ cells co-expressed TPBG, indicating that EGFP reflected endogenous *Tpbg* expression in *Tpbg*-EGFP mice. Further examination revealed that EGFP signal and immunoreactivity of TPBG were detected at in the floor plate (FP) of the midbrain from E9.5 (Fig. 1a). The expression was also detected in the dorsal side of the diencephalic region, and it eventually became localized at the cortical hem (at E12.5, Fig. 1a). However, we focused on its expression in the VM region to investigate its potential involvement in the differentiation of mDA neurons. Strong expression was maintained until E12.5, at which mDA neurogenesis reaches at peak. To identify the cell types that express *Tpbg* in the FP, we labeled the EGFP-expressing tissue with antibodies against several mDA markers. At E11.5, the sagittal sections of the developing mouse brain highlighted EGFP expression throughout the rostro-caudal axis of the VM. This expression pattern overlapped with immunoreactivity to LMX1A and FOXA2, specific markers for mDA precursors and FP, respectively, suggesting that *Tpbg*-expressing cells are a subset of mDA neuron progenitors (Fig. 1b). Further examination of sagittal sections at E12.5 showed that a subset of EGFP^+^ cells co-expressed NURR1 and/or TH, conventional markers for mDA neurons (Fig. 1b). Most of the rostral population of EGFP^+^ cells exhibited co-labeling with mDA markers; however, few cells overlapped with these markers in the caudal midbrain (Fig. 1b). In the coronal sections, EGFP^+^ cells were initially concentrated at the medial part of the ventral midline, and they overlapped with FOXA2- and LMX1A-expressing mDA domains through the length of the VM at E11.5 and E12.5 (Fig. 1c). During this period, the neuroepithelium is divided into three layers, according to the developmental stage of the mDA lineage cells that compose each domain: the ventricular zone (VZ), intermediate zone (IZ), and marginal zone (MZ) (Supplementary Fig. 1a). EGFP expression was detected in SOX2^+^ progenitor cells of the VZ, NURR1^+^ mDA neuroblasts of the IZ, and TH^+^ postmitotic mDA neurons of the MZ (Supplementary Fig. 1b), suggesting that *Tpbg* is expressed in mDA lineage cells throughout the developmental stages of the rostral VM. Between E12.5 and E15.5, which is the migration phase of mDA progenitors, EGFP^+^ cells exhibited radially and tangentially oriented bipolar morphology in the mDA domain (i.e., IZ and MZ) (Fig. 1d). In addition, most EGFP^+^ cells in that domain overlapped with the NURR1^+^ mDA neuroblasts, while a fraction of them were also positive for TH (Supplementary Fig. 1c). These results indicate that *Tpbg* is initially expressed in the mDA progenitor population, and its expression is sustained in postmitotic mDA neurons. Notably, such characteristics of EGFP^+^ cells, including cellular composition, distribution, and morphology, were evident in the rostral part but not as much as in the caudal part of the VM region (Fig. 1). These features during a critical period of mDA neurogenesis were similar to those of the developing mDA cell population destined for the SNc^25,26^.

### *Tpbg* remains expressed in the dopaminergic subsets of adult mouse ventral midbrain

**Fig. 2:**
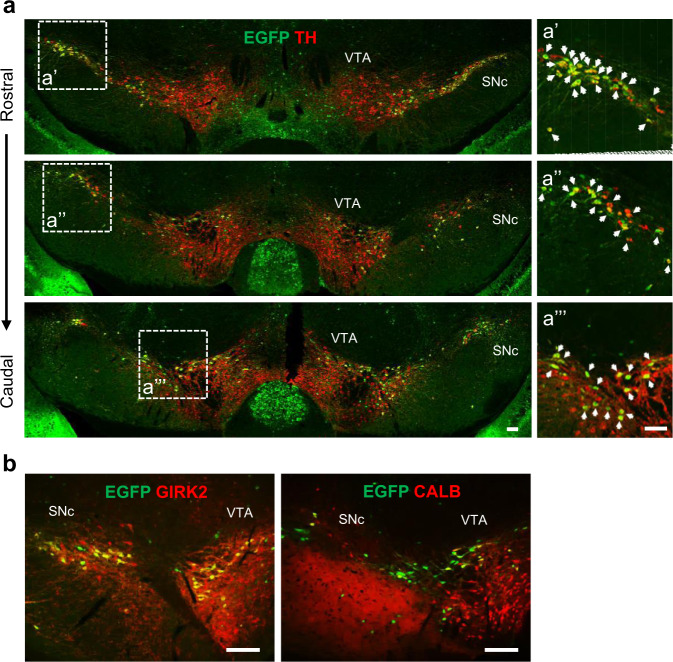
Analysis of TPBG expression in the adult mDA subsets. **a** Immunohistochemical staining showing EGFP expression in the SNc and VTA of adult mice in a coronal manner. In the high magnification images (a’, a”, and a”’), the white arrows indicate EGFP (*Tpbg*) co-expressing TH^+^ mDA neurons. **b** EGFP was mostly co-expressed with GIRK2 and partially with CALB in the mDA subsets (Scale bars: 100 μm).

We examined whether *Tpbg* expression was maintained in the mDA domains of the adult VM. In situ hybridization data from the Allen Institute database (https://mouse.brain-map.org/) showed that *Tpbg* transcripts were detected in the same region (SNc) expressing *Girk2* and in the VTA region marked by *Calbindin1* (*Calb*) on postnatal day 56 (Supplementary Fig. 2). Consistent with the mRNA expression pattern, our immunohistochemical analysis showed that EGFP was detected in most TH-positive neurons in the SNc (GIRK2^+^) and in a small portion of the VTA (CALB^+^) (Fig. 2a, b).

Collectively, these data indicate that *Tpbg* was expressed in the mDA lineage cells of the mouse VM region during embryonic development, and its expression was maintained in mDA neurons in the adult mouse brain.

### Genetic ablation of *Tpbg* induces loss of midbrain dopaminergic neurons, accompanied by axonal defects in aged mice

Histological analysis of *Tpbg*-EGFP mice revealed that *Tpbg* was expressed in the mDA domain in the developing embryonic brain, as well as in the adult brain. These results prompted us to hypothesize that *Tpbg* may not only be involved in the development of mDA neurons but also be required for the maintenance of mDA systems later in life. To answer this question, we explored the neuroanatomical and neurochemical changes in mDA systems using *Tpbg* knockout (KO) mice.

The ablation of the *Tpbg* gene in the *Tpbg* KO brain was confirmed by PCR-based genotyping and western blotting (Supplementary Fig. 3). Although previous studies using the same mouse line as ours (i.e., Tpbg^tm1Lex^) did not report abnormal brain structure in their colony of *Tpbg* KO mice, we observed that a few subjects exhibited hydrocephalic phenotypes (e.g., unusually large head size and a dome-shaped head) in specific generations at F3 and their progenies (F4~), consistent with other studies using different mouse lines (Tpbg^tm1Plst^)^20,27,28^. We attributed the random occurrence of the hydrocephalic phenotype in our experiment to the difference in the genetic background of the mice (C57Bl/6 for the previous study demonstrating the hydrocephalus vs. C57Bl/6 × 129/Sv in this study). To focus on the phenotype in the midbrain, we excluded the colony exhibiting the hydrocephalic phenotype in their genealogy according to histological evaluation. Histological examination revealed that the overall structure of *Tpbg* KO mouse brains was comparable to that of wild-type (WT) counterparts except for structural abnormalities in the hippocampus of *Tpbg* KO mice (Supplementary Fig. 4).

**Fig. 3:**
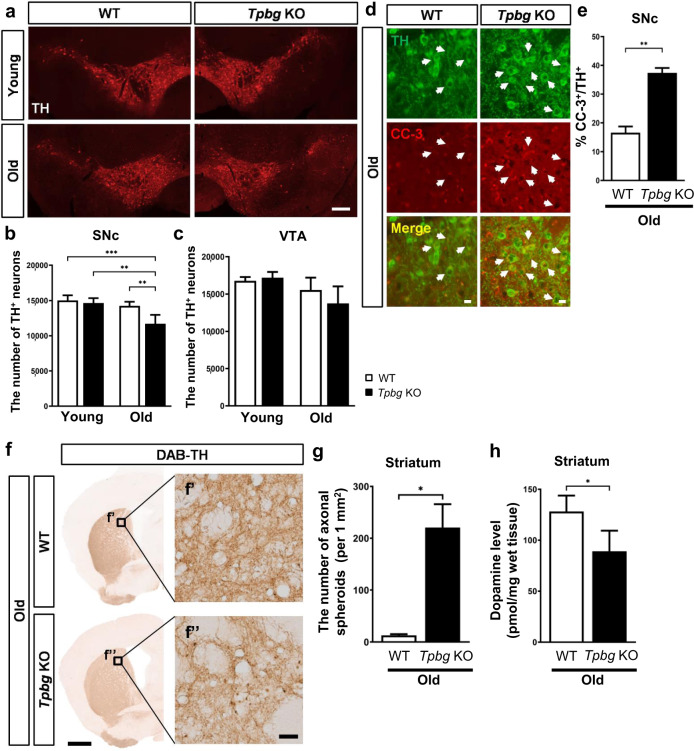
Investigation of neuroanatomical and neurochemical changes in the mDA system. **a** Representative images of immunohistochemical staining for TH in the VM of young (3–4 months) and old (12–14 months) WT and *Tpbg* KO mice (Scale bar: 250 μm). **b–c** Quantification of the number of TH^+^ mDA neurons in the SNc (**b**) and VTA (**c**) of WT and *Tpbg* KO mice at young (WT, *n* = 4; KO, *n* = 4) and old age (WT, *n* = 5; KO, *n* = 5). Bars represent the number of TH^+^ neurons in the mDA subsets of each group (** *P* < 0.01, *** *P* < 0.001; two-way ANOVA with Sidak’s multiple comparison test). **d** Representative images of immunohistochemical staining for TH and cleaved CASPASE-3 (CC-3) in the SNc of old WT and *Tpbg* KO mice. White arrows indicate CC-3-co-expressing TH^+^ neurons (Scale bars: 25 μm). **e** Quantification of the number of CC-3^+^ cells among TH^+^ neurons in the SNc of old WT (*n* = 5) and *Tpbg* KO (*n* = 5) mice. Bars represent the percentage (%) of CC-3^+^ cells among TH^+^ neurons in the SNc of *Tpbg* KO mice and WT counterpart (** *P* < 0.01; Mann–Whitney test). **f** Representative images of DAB staining for TH^+^ fiber in the striatum of old WT and *Tpbg* KO mice. High-magnification images (f’ and f”) show that TH^+^ spheroid-like structures were abundant in the striatum of the aged *Tpbg* KO mice [Scale bar: 1 mm (Low magnification); 25 μm (high magnification)]. **g** Quantification of the number of axonal spheroids in the striatum of old WT (*n* = 4) and *Tpbg* KO (*n* = 4) mice (* *P* < 0.05; Mann–Whitney test). **h** Dopamine levels in the striatum of old WT (*n* = 5) and *Tpbg* KO (*n* = 4) mice were measured using ELISA and normalized to the amount of total tissue (mg) (* *P* < 0.05; Mann–Whitney test). Data are represented as the mean ± SD (**b**, **c**, **e**, and **h**) or mean ± SEM (**g**).

There was no significant difference in the number and distribution of mDA neurons between WT and *Tpbg* KO mice at E18.5 (data not shown), indicating that the loss of *Tpbg* did not alter the formation of mDA subsets during embryonic development. To investigate whether genetic ablation of *Tpbg* affects the maintenance of mDA neurons later in life, the number of TH-positive (TH^+^) mDA neurons was quantified in the SNc and VTA of adult *Tpbg* KO and WT mice at young and old ages (Fig. 3a–c). The number of TH^+^ mDA neurons in the midbrain of young mice showed no differences between the two genotypes (Fig. 3b, c). At old age, however, the total number of TH^+^ neurons in the *Tpbg* KO mice was lower than that in WT counterparts, and the reduction was significantly greater in the SNc than in the VTA (Fig. 3b, c). *Tpbg* hemizygous (Hem) mice did not show a significant decrease in the number of TH^+^ cells in either the SNc or VTA (data not shown). Consistent with this result, we detected an increased number of TH^+^ cells positive for cleaved CASPASE-3 (CC-3) in the SNc (Fig. 3d, e), indicating that the decrease in the number of TH^+^ cells in aged *Tpbg* KO mice might be a result of apoptosis. The selective vulnerability of mDA neurons to *Tpbg* ablation correlates with the enriched expression of *Tpbg* in the SNc (Supplementary Fig. 2 and Fig. 2b) and the previous result showing differential *Tpbg* expression between SNc and VTA^29,30^. Because the TH^+^ neurons in the SNc were diminished in number, we explored whether the functional connectivity of the mDA neurons to the axonal projection target is affected by *Tpbg* ablation. In particular, neurochemical changes in the striatum were assessed. TH^+^ fiber density in the striatum was not significantly different between *Tpbg* KO mice and WT controls (Supplementary Fig. 5). However, we observed spheroidal dystrophic terminals in the TH^+^ nerve fibers in the striatum of aged *Tpbg* KO mice (Fig. 3f, g). In accordance with the abnormal mDA nerve terminals, striatal dopamine contents in aged *Tpbg* KO mice were significantly reduced by 30% compared to that in the age-matched WT control (128.24 ± 15.6 pmol/mg in WT vs. 89.21 ± 20.23 pmol/mg in *Tpbg* KO) (Fig. 3h). Scattered TH^+^ cell bodies were also observed in the striatum of *Tpbg* KO mice (Supplementary Fig. 6), a likely compensatory mechanism for the dystrophic nerve terminal, as demonstrated by previous studies of the striata from PD brains^31,32^. Collectively, these data indicate that *Tpbg* ablation led to the loss of mDA neurons in the SNc with age, accompanied by impairment of dopaminergic innervation to the striatum in the mouse brain.

### Pathophysiological features of Parkinson’s disease were observed in the ventral midbrain of *Tpbg* knockout mice at old age

**Fig. 4:**
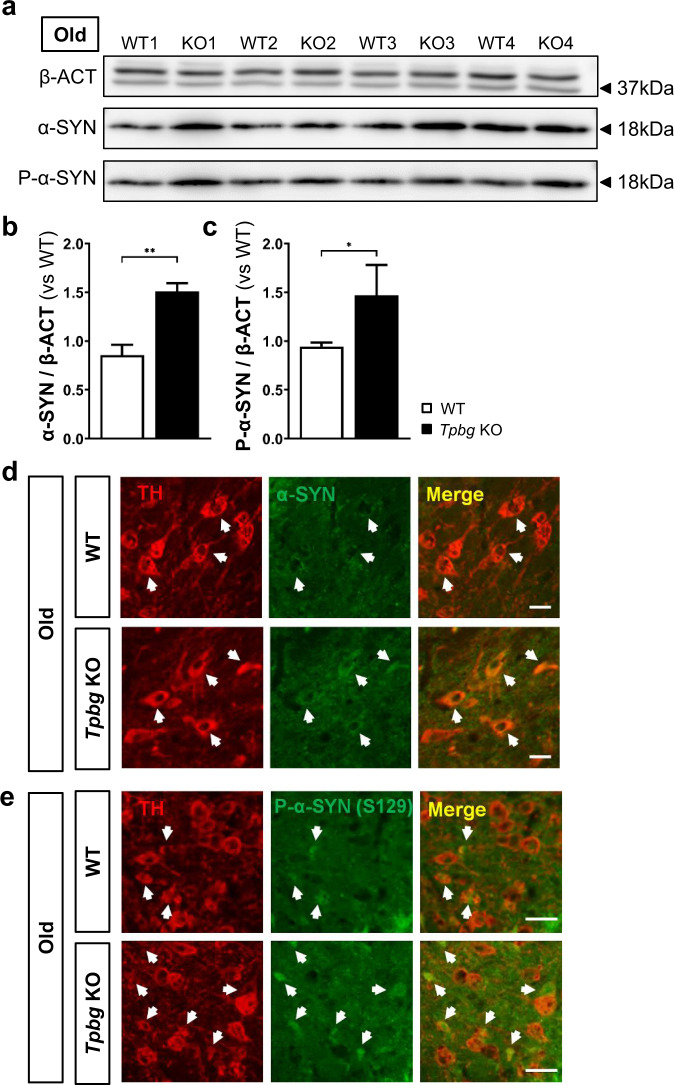
Investigation of accumulation of α-synuclein in the SNc of aged *Tpbg* KO mice. **a** Representative images of western blotting for α-synuclein (α-SYN) and serine129-phosphorylated α-synuclein (P-α-SYN) in the SNc of WT and *Tpbg* KO mice at old age. Molecular size markers are shown in kilodaltons (kDa). Un-cropped images of blots are shown in the Supplementary Fig. 12b. **b–c** Quantification of band intensity for α-SYN (**b**) and P-α-SYN (**c**) in the SNc of WT (*n* = 4) and *Tpbg* KO (*n* = 4) mice at old age. Bars represent α-SYN and P-α-SYN levels normalized to β-actin (β-ACT) levels, relative to the WT control (WT was set at 1.0; * *P* < 0.05, ** *P* < 0.01; Mann–Whitney test). **d–e** Representative images of immunohistochemical staining for α-SYN (**d**) and P-α-SYN (**e**) in the SNc of WT and *Tpbg* KO mice at old age. White arrows indicate α-SYN- or P-α-SYN-co-expressing TH^+^ mDA neurons (scale bar: 20 μm).

**Fig. 5:**
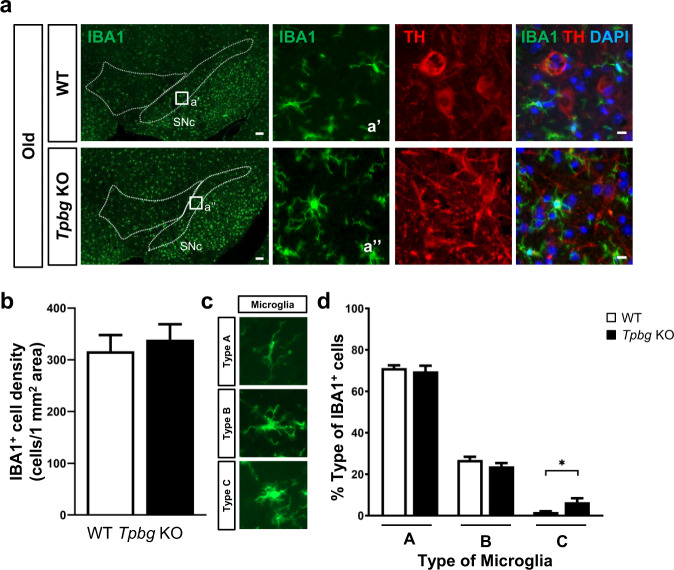
Analysis of microglial activation in the SNc of aged *Tpbg* KO mice. **a** Representative images of immunohistochemical staining for IBA1^+^ microglia in the VM of WT and *Tpbg* KO mice at old age. Low magnification images illustrate the distribution of IBA1^+^ microglia throughout the VM, whereas the adjacent higher magnification images (a’ and a’’) show the morphology of microglia in the SNc [scale bar: 100 μm (low magnification); 20 μm (high magnification)]. **b** Quantification of the total IBA1^+^ cell density (the total number of IBA1^+^ cells per unit area of 1 mm^2^) in the SNc. Data is represented as the mean ± SD (WT, *n* = 5; KO, *n* = 5; not significant, *P* > 0.05; Mann–Whitney test). **c** Representative images of three types of IBA1^+^ nigral microglia as a function of their degree of activation. **d** Classification and quantification of IBA1^+^ microglia into three types. Values represent the percentage of each type of microglia in the SNc and are expressed as the mean ± SEM (* *P* < 0.05; Mann–Whitney test).

Reduction in the number of TH^+^ cells in the SNc and striatal dopamine levels prompted us to further investigate whether genetic ablation of *Tpbg* induces the pathophysiological features of PD in mice. One of the pathological hallmarks of PD is the accumulation of abnormal α-SYN, which may contribute to Lewy-like inclusion formation and lead to neuroinflammation. To address this, we examined the presence of α-SYN and S129-phosphorylated α-SYN (P-α-SYN) in the SNc of aged *Tpbg* KO mice using western blotting. The results revealed that the band intensity for α-SYN and P-α-SYN significantly increased in the SNc of aged *Tpbg* KO mice compared with that in the SNc of age-matched WT mice (Fig. 4a–c). Furthermore, immunohistochemical analysis revealed that TH^+^ cells were more frequently labeled with antibodies for both α-SYN and P-α-SYN in aged *Tpbg* KO mice than in WT counterpart (Fig. 4d, e). However, the number of Lewy-like aggregates in the SNc was not significantly different between the WT and *Tpbg* KO mice (Supplementary Fig. 7). Next, to investigate whether the accumulation of α-SYN is accompanied by the sign of neuroinflammation, we performed immunohistochemical analysis with an antibody targeting microglia (anti-IBA1) (Fig. 5a). Total IBA1^+^ cell density (the number of IBA1^+^ microglia per unit area of 1 mm^2^) showed no significant difference between the two genotypes (Fig. 5b). However, upon analyzing the results on the basis of their morphological characteristics (Fig. 5c), we found that the proportion of IBA1^+^ cells with enlarged cell bodies and fewer short and thick branches (type C), which are the morphological features of activated microglia^33,34^, were significantly higher than that of the simply ramified IBA1^+^ cells (type A and B) in the SNc of the aged *Tpbg* KO mice (Fig. 5d). This result suggests that the SNc of aged *Tpbg* KO mice may present higher levels of neuroinflammation than that of age-matched WT counterpart.

Collectively, our results show that genetic ablation of *Tpbg* results in pathological changes in the VM of aged mice, which may create an unfavorable environment for the survival of mDA neurons.

### Aged *Tpbg* knockout mice display motor deficits in nigrostriatal pathway-sensitive behavioral tests

**Fig. 6:**
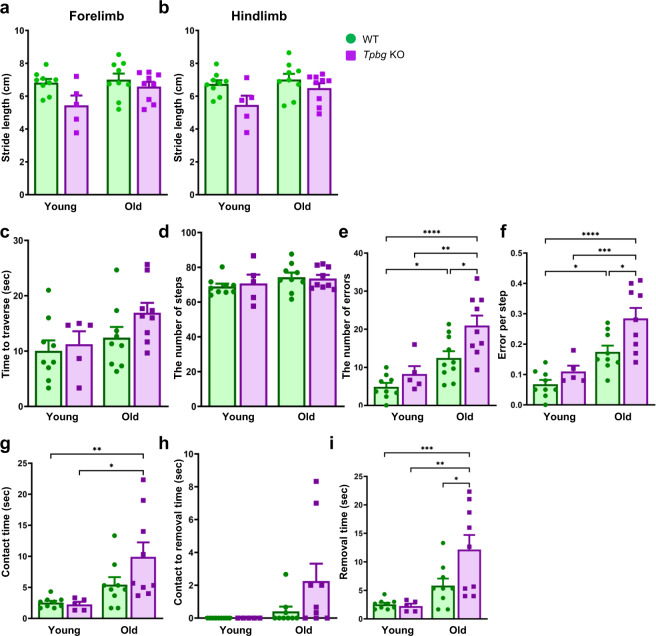
Assessment of sensorimotor tests that are sensitive to alterations in the nigrostriatal dopaminergic system. Behavioral examination of WT and *Tpbg* KO mice at young (WT, *n* = 9; KO, *n* = 5) and old age (WT, *n* = 9; KO, *n* = 9) was performed as follows: **a–b** Gait patterns were measured as forelimb (**a**) and hindlimb (**b**) stride lengths. **c–f** Motor performance and coordination were assessed using the challenging beam travel test, as measured by the time to traverse (**c**), the number of steps (**d**), the number of errors (**e**), and errors per step (**f**). **g–i** Sensorimotor function was assessed by measuring the time to contact (**g**), contact to removal time (**h**), and time to removal (**i**) in the adhesive-removal test. Data is represented as the mean ± SEM (* *P* < 0.05, ** *P* < 0.01, *** *P* < 0.001, **** *P* < 0.0001; two-way ANOVA with Sidak’s multiple comparison test).

Since *Tpbg* KO mice show pathological features reminiscent of PD, we questioned whether the ablation of *Tpbg* results in the development of PD-like motor symptoms. To answer this question, we evaluated motor function using a battery of behavioral tests sensitive to alterations in the nigrostriatal dopaminergic system. When young and old *Tpbg* KO and WT mice were weighed before behavioral testing, no significant difference was observed with respect to their genotypes and ages (Supplementary Fig. 8), confirming that the body weight could be excluded from factors affecting behavioral phenotype. Gait analysis was performed using the footprint method to quantify the potential difference in walking patterns. The results revealed that there was no significant difference in all parameters of gait analysis, including stride length, stride width, and intra-step distance among different genotypes (Fig. 6a, b and Supplementary Figs. 9, 10a, b). A challenging beam travel test was conducted to further examine motor performance and coordination. Again, no significant difference was observed in the time to traverse and the number of steps taken while traversing the beam among different genotypes (Fig. 6c, d and Supplementary Fig. 10c, d). However, *Tpbg* KO mice made significantly more errors and errors per step while traversing the beam than old WT mice with the number of erroneous steps increasing with age (Fig. 6e, f). Consistent with histological evidence, young *Tpbg* KO and old *Tpbg* Hem mice did not show significant motor deficits (Figs. 3b, 6c–f and Supplementary Fig. 10c–f). These results suggest that *Tpbg* KO mice exhibit deficits in motor performance and coordination only at old age. Lastly, to assess motor response to sensory stimuli, we measured the time to detect a stimulus and the time taken to remove it using the forepaws. In young mice, there were no significant differences in the ‘time to respond’ and ‘time to make a contact with sensory stimuli’ between the genotypes (Fig. 6g–i). On the contrary, the time taken to make contact with the sensory stimulus and the time taken to remove it after contact increased in aged *Tpbg* KO mice, although the latter was not statistically significant compared with age-matched WT and *Tpbg* Hem mice (Fig. 6g, h and Supplementary Fig. 10g, h). More importantly, the total time elapsed between making contact with the stimulant and removing it was significantly longer for aged *Tpbg* KO mice than for either young *Tpbg* KO (age controls) or aged WT mice (genotype controls) (Fig. 6i). These results indicated that aged *Tpbg* KO mice showed mild impairment in both paw and snout sensitivity and dexterity; however, the cumulative effect of both produced a significant defect in sensorimotor function.

Together with biochemical and histological evidence, impaired motor performance and sensorimotor dysfunction observed in aged *Tpbg* KO mice strongly suggests that *Tpbg* is a candidate gene associated with the development of PD-like phenotypes in mice.

### In silico prediction identified potential partners interacting with TPBG that are relevant to Parkinson’s disease pathogenesis

**Fig. 7:**
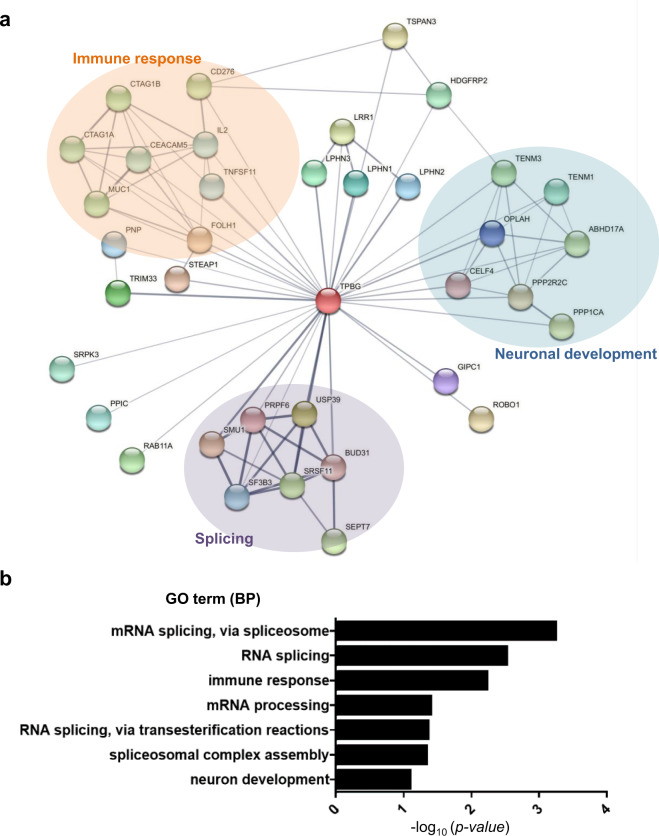
Identification of putative functional partners and biological function of TPBG based on bioinformatic tools. **a** The protein–protein interaction (PPI) network analysis of putative functional partners of TPBG using the STRING database. Three distinct functional clusters were identified within the PPI network using Cytoscape and MCODE. Each node is representative of a protein, and the connecting lines represent interactions between proteins. Each cluster is a set of highly-connected nodes and is illustrated in a discrete color: splicing, purple; immune response, orange; and neuronal development, blue. **b** Functional enrichment analysis using GeneOntology (GO) for TPBG-interacting partners, based on DAVID. The words on the left indicate the enriched GO term in biological process (BP).

To explore the mechanism underlying the PD-like phenotypes of *Tpbg* KO mice, we searched for proteins that may interact with TPBG using STRING, a biological database for visualizing protein–protein interaction (PPI) networks. Initially, we obtained 36 proteins from STRING that are expected to interact with TPBG with a cutoff value of 0.4 (medium interaction confidence)^35^. A network analysis of the proteins interacting with TPBG, using a clustering algorithm (MCODE)^36^ identified three major clusters involved in the biological functions of splicing, immune response, and neuronal development (Fig. 7a). Furthermore, GeneOntology (GO) enrichment analysis using the 36 proteins revealed the same three functional groups enlisted at the top of the list (Fig. 7b)^37,38^. Surprisingly, many of the TPBG-interacting partners are involved in splicing-related functions. The malfunction of splicing has been implicated in neurodegenerative diseases, including PD^39–41^. In particular, WD40 repeat-containing protein SMU1 (SMU1), serine/arginine-rich splicing factor 11 (SRSF11), ubiquitin-specific peptidase 39 (USP39), protein BUD31 homolog (BUD31), splicing factor 3B subunit 3 (SF3B3), and pre-mRNA-processing factor 6 (PRPF6) play a role in pre-mRNA splicing as components of the splicing machinery (spliceosome), and their aberrant functioning is highly implicated in PD^41–44^.

The enrichment of genes related to immune response, such as CD276 antigen (CD276), interleukin-2 (IL2), tumor necrosis factor ligand superfamily member 11 (TNFSF11), purine nucleoside phosphorylase (PNP), and mucin-1 (MUC1), was also intriguing. Although previous studies have implicated the innate and adaptive immune system in PD pathobiology and disease severity^45–47^, there is limited evidence linking TPBG to the immune response. Thus, our data cast an interesting possibility that the absence of functional TPBG may cause PD pathogenesis through the modulation of multiple genes related to immune responses.

Neuron development was the third on the list of biological functions of TPBG interactors from network analysis and GO enrichment analysis. Several genes enriched in this cluster have been involved in synaptic transmission and connectivity of neurons. For example, PPP1CA and PPP2R2C, subunits of protein phosphatase 1 and 2 (PP1 and PP2), are important for the regulation of dopamine release; they act on the SNARE complex and synaptic plasticity in the dopaminergic synapse^48–50^. Interaction with these factors was likely lost in *Tpbg* KO mice, which might be responsible for reduced dopamine content in the striatum of *Tpbg* KO mice (Fig. 3h).

Several putative TPBG-interaction partners have also drawn our attention because they are involved in other cellular processes related to PD pathology. First, both tripartite motif-containing 33 (TRIM33), an E3 ubiquitin-protein ligase, and USP39, a deubiquitinating protein, play roles in the ubiquitin proteasome system (UPS). The UPS is an intracellular protein degradation system responsible for the majority of protein turnover within the cell^51,52^. Dysfunction of the UPS has been strongly implicated in PD pathogenesis^53–55^ and α-SYN clearance^56,57^. Therefore, it is plausible to speculate that the dysfunction of USP39 due to loss of TPBG function might be linked to the accumulation of α-SYN (Fig. 4). Second, the three isoforms of latrophilin (LPHN1, LPHN2, and LPHN3) have been identified as interaction partners of TPBG. Previous studies have implicated LPHNs in the dopamine metabolic system and dopamine neurotransmission^58,59^ thus, the reduced dopamine content in the striatum of *Tpbg* KO mice might be caused by the misregulation of LPHNs.

For the experimental validation of our hypothesis suggested from in silico analysis, we conducted RNA-seq analysis of the SNc of aged *Tpbg* KO mice and WT counterpart (Supplementary Fig. 11). Since the analysis was to confirm our proposed hypothesis, we investigated whether the genes involved in splicing and neuronal development were captured in the RNA-seq analysis. For this analysis, we performed gene set enrichment analysis (GSEA) using the KEGG and GO databases. As shown in Supplementary Fig. 11b–d, the expression patterns of the genes involved in spliceosome and neuronal development were significantly different between the *Tpbg* KO mice and WT counterpart, and those of the genes involved in PD were also significantly different. In the enrichment analysis, we were unable to capture specific immune responses because of the large number of child terms of immune response in GO and a large number of genes involved in immune response-related terms. In general, protein-network analysis is useful for identifying local and specific cellular processes for in-depth study, while enrichment analysis is useful for obtaining a broad insight into global phenomena. Therefore, further studies on specific immune responses are required for elucidating the association between immune response and *Tpbg* ablation. Despite of the missing of immune response in GSEA analysis results, the additional analysis results represent that *Tpbg* is implicated in PD via splicing and neuronal development processes. In addition, the similarities in histology and behavior between PD and *Tpbg* KO mice provide further evidence that *Tpbg* is a candidate gene associated with PD.

## Discussion

This study demonstrates that *Tpbg* is specifically expressed in the VM of the developing embryonic brain and adult midbrain in mice. *Tpbg*-expressing cells initially arise at the medial FP (FOXA2^+^LMX1A^+^) and subsequently migrate out to the MZ along a course similar to that of the differentiating mDA progenitors (NURR1^+^TH^+^). Eventually, they become restricted to the rostro-lateral domain of the VM and remain at the same position (both SNc and VTA) for the lifetime. These developmental features of *Tpbg*-expressing cells suggest that *Tpbg* may be intimately involved in the development and function of mDA neurons.

Given the previous reports demonstrating that TPBG regulates Wnt and CXCR4 chemokine signaling^12,19,20^, it was surprising that neither late embryos of *Tpbg* KO mice nor young *Tpbg* KO mice showed any alteration in the distribution or number of mDA neurons. The essential roles of Wnt signaling have been well characterized in the differentiation of mDA neurons^60^. Thus, the absence of developmental defects suggests that TPBG may not be actively engaged with the Wnt signaling pathway in the development of mDA neurons. Additionally, CXCR4/CXCL12-mediated chemokine signaling was found to be required for the radial migration of mDA lineage cells^61^. However, TPBG may not exert a critical influence on the CXCR4/CXCL12 signal in mDA neuronal migration. Alternatively, the migratory defect of mDA lineage cells in the absence of a functional CXCR4/CXCL12 signal is transient and limited to the initial radial migratory phase^26^. Furthermore, numerous chemokine signals are involved in the migration and axon guidance of mDA lineage cells^62^. Therefore, it is speculated that multiple cellular signals might complement each other to rescue the migration defect caused by the absence of TPBG. Lastly, the phenotype of a specific gene deficiency has often been differentially presented in mice with different genetic backgrounds^63,64^ thus, we cannot exclude the possibility that the lack of developmental defects is only restricted in the mouse strain used in this study.

Although there was no noticeable phenotype at the late embryonic stage and young age of *Tpbg* KO mice, we observed several PD-related pathological signs at old age. Aged *Tpbg* KO mice exhibited a significant reduction in the number of TH^+^ cells in the SNc, likely due to apoptosis, along with α-SYN accumulation, increased microglial activation, and a decline in striatal dopamine content. Interestingly, the loss of TH^+^ cells was more pronounced in the SNc than in the VTA. The molecular basis behind this selective loss is unclear; however, considering that TPBG was preferentially expressed in the SNc than in the VTA, ablation of *Tpbg* seems to have a more negative impact on the survival of TH^+^ cells in the SNc than in the VTA. Consistent with this idea, previous reports have demonstrated that differential gene expression between the SNc and VTA impacts the selective vulnerability of mDA neurons in PD^29,30^.

Indeed, these cellular phenotypes were later accompanied by their PD-like behavioral phenotypes, albeit somewhat mild, and we observed impairments in motor performance and/or coordination and somatosensory function in aged *Tpbg* KO mice in the challenging beam travel test and adhesive removal test but no alteration in gait performance. However, motor dysfunction correlated well with the extent of dopaminergic loss: aged *Tpbg* KO mice showed 18% less mDA neurons in the SNc and 30% lower dopamine content in the striatum compared with WT counterparts. Since previous clinical studies have estimated that at least 30% of mDA neuronal cell bodies were lost in the SNc and that dopamine contents were decreased by 68–82% in the striatum at the onset of motor symptoms in PD patients^65^, such mild defects in motor performance and coordination appear reasonable.

Our in silico analysis predicted that TPBG interacts with various protein partners involved in splicing, immune response, neuronal development, protein homeostasis, and several other minor cellular functions, including UPS and dopamine metabolism. Furthermore, our unbiased transcriptomic analysis substantiated the in silic*o* prediction by demonstrating alterations in gene expression related to similar biological processes. Alteration in the expression of certain genes appeared to critically contribute to PD-like phenotypes, as a plethora of evidence has implicated aberrant splicing and unbalanced protein homeostasis in the pathophysiology of PD^44,52^. Most importantly, the enrichment of PD-related genes strongly supports the pathological phenotypes that we observed in the histological and behavioral analyses. Nonetheless, it is still obscure whether *Tpbg* deficiency directly causes such transcriptomic alteration, because this result might also have been an indirect consequence of the pathological changes manifested in the SNc of aged *Tpbg* KO mice; that is, up-regulation of genes involved in PD may reflect the occurrence of mDA degeneration in aged *Tpbg* KO mice. A more detailed molecular investigation will be required to determine the causal relationship between ablation of TPBG and the altered expression of the genes involved in PD pathogenesis.

**Fig. 8:**
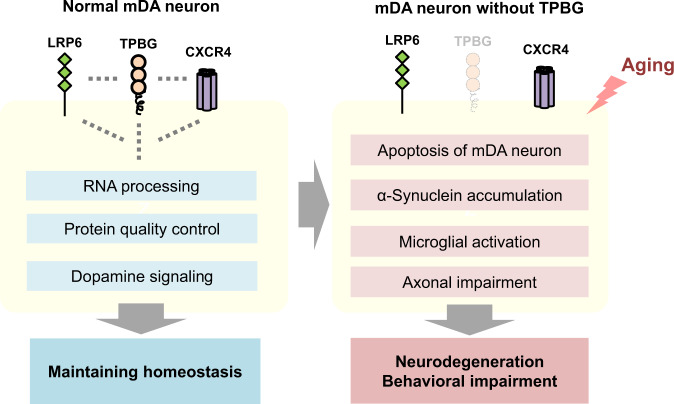
A putative mechanism of how TPBG may contribute to PD pathogenesis. Under normal conditions, TPBG may be involved in RNA processing, protein quality control system, and dopamine signaling, contributing towards maintaining mDA homeostasis as a “moderator”. In the context of environmental triggers (i.e., aging), however, lack of TPBG function may result in disruption of the cellular mechanisms moderated by TPBG and induce pathological changes resulting in unfavorable environment for functioning and maintaining mDA system, eventually leading to mDA degeneration and behavior impairment. Blue squares indicate the biological process in which TPBG may be involved, according to in silico and transcriptomic analysis. Red squares indicate the pathophysiological features of PD developed in the brain of *Tpbg* KO mice with age. mDA, midbrain dopaminergic.

On the basis of these findings, we propose a hypothetical model in which TPBG plays a role in maintaining the homeostasis of mDA neurons while interacting with protein factors involved in RNA processing, protein quality control, and dopamine metabolism (Fig. 8). Given such multifaceted involvement of TPBG in various cellular mechanisms and its putative existence on the plasma membrane of mDA neurons, TPBG may function as a “moderator” that modulates the proper functioning of diverse cellular mechanisms while monitoring both extracellular and intracellular environments. For example, as a potential regulator of Wnt signaling, TPBG may assist Wnt signaling in playing an important role in synaptic maintenance and function in the adult nervous system^66^ or may affect the interaction of LRP6 with leucin-rich repeat kinase 2 (LRRK2), a factor responsible for familial PD^67^. The ability to interact with CXCR4/CXCL12 may also influence synaptic function and neuronal survival in the adult brain, as the alteration of CXCR4/CXCL12 has been involved in the impairment of synaptic transmission and neuro-regeneration in mice^68^. In such a scenario, failure in the ability of *Tpbg* to function would not be immediately disastrous; however, the interconnection of the cellular mechanisms moderated by *Tpbg* could eventually disintegrate and become pathological. Subtle cellular and behavioral phenotypes of *Tpbg* KO mice, which are observed only in old age, suggest that *Tpbg* mutation is unlikely to be a causative factor of PD; instead, it may act as a risk factor that increases the odds of disease onset triggered by other critical factors such as aging.

To our knowledge, this is the first study to investigate the potential correlation between *Tpbg* and PD pathogenesis. Although further functional studies should be conducted to elucidate the detailed molecular mechanisms of how TPBG interacts with its partners and how the interaction contributes to the homeostasis of the mDA system, our study suggests that *Tpbg* is a potential candidate gene associated with PD and provides a new insight into the molecular mechanism underlying the pathophysiology of PD. Lastly, previous genome-wide association studies have never identified *TPBG* as a candidate gene for PD; thus, the involvement of *Tpbg* in human disease needs to be verified through further genetic studies.

## Methods

### Animals

#### *Tpbg*-EGFP reporter line

*Tpbg*-EGFP mice on a mixed FVB/N and Crl:CD1(ICR) background [strain Tg(TPBG-EGFP) NJ116 Gsat^69^, MMRRC, archived at the generation N1F2] were purchased as cryo-preserved spermatozoa and recovered on a C57BL/6N (Orient Bio, Seongnam, South Korea) background at the Yonsei Biomedical Research Institute. F1/F2 heterozygous mice were backcrossed with C57BL/6N mice, and F3/F4 WT and heterozygous mice were sib-mated to generate F5 heterozygous mice. In all experiments, female heterozygous mice were used, which were obtained by intercross breeding of WT and heterozygous mice of the following age and number of mice: 9 embryos between E9.5 and E15.5 (E9.5, *n* = 1; E10.5, *n* = 2; E11.5, *n* = 2; E12.5, *n* = 2; E13.5, *n* = 1; E15.5, *n* = 1) and two adult mice (9 months old).

#### *Tpbg* knockout line

*Tpbg* KO mice on a mixed C57BL/6 and 129/SvEvBrd background (strain Tpbgtm1Lex^70^, MMRRC, archived at the sib-mated generation F4) were purchased as cryopreserved spermatozoa and recovered on C57BL/6N mice. *Tpbg*^+/−^ (*Tpbg* Hem) progeny were intercrossed each generation thereafter to generate F5 *Tpbg*^+/+^ (WT), *Tpbg* Hem, and *Tpbg*^−/−^ (*Tpbg* KO) mice. In all experiments, female WT, *Tpbg* Hem, and *Tpbg* KO mice were used, which were obtained by intercross breeding of *Tpbg* Hem mice as follows: 47 adult mice (young age, 3–4 months old; WT, *n* = 9; *Tpbg* KO, *n* = 5; old age, 12–14 months old; WT, *n* = 14, *Tpbg* KO, *n* = 13; *Tpbg* Hem, *n* = 6). All experimental procedures for this study were approved by the Institutional Animal Care and Use Committee of the Yonsei University Health System. Mice were maintained in a specific pathogen-free barrier facility with a 12 h light/dark cycle. The mouse genotypes were characterized using a PCR-based strategy. For genotyping, tails of pre- or postnatal mice were cut at 1 mm from the end, and genomic DNA was obtained using Direct PCR Lysis Reagent (Viagen Biotech, Inc., LA, CA, USA) according to the manufacturer’s instructions. Polymerase chain reaction (PCR) was performed using a PCR thermal cycler (Applied Biosystems Geneamp 2720), Emerald Amp^®^ GT PCR Master Mix (TAKARA Bio Inc., Shiga, Japan), and primers listed in Supplementary Table 1.

### Histological analysis

For embryo analysis, the mice were allowed to mate overnight, and if the vaginal plug was observed at noon of the following day, it was considered E0.5. Pregnant mice were anesthetized with a mixture of Zoletil and Rompun, and the uterine horns were dissected. The embryos were removed from the uterine horns in ice-cold phosphate-buffered saline (PBS), fixed in 4% paraformaldehyde (PFA) for 4 h to overnight, and cryoprotected in 15–30% sucrose-PBS solution. The cryoprotected tissue samples were embedded in FSC 22^®^ compound (Leica, Nußloch, Germany), serially sectioned with a cryostat (Thermo Scientific, Waltham, MA, USA) at 14 μm thickness, and attached onto glass slides. For analysis of the adult brain, the mice were anesthetized with 15% urethane solution and transcardially perfused with 0.9% saline solution followed by 4% PFA. The brains were dissected, post-fixed overnight, and cryoprotected in 30% sucrose-PBS solution. The cryoprotected tissue samples were embedded in FSC 22^®^ compound, serially sectioned with a cryostat at 16 μm thickness, and stored in a tissue stock solution at −20 °C. For immunohistochemistry, the sections were pre-incubated in 0.3% Triton X-100 and 3% bovine serum albumin (BSA)-PBS solution (blocking solution) for 1 h at 25 °C followed by overnight incubation at 4 °C with primary antibodies. After washing with PBS, the sections were incubated for 2 h at 25 °C with the appropriate fluorescence-tagged secondary antibodies and mounted in a mounting medium containing 4,6-diamidino-2-phenylindole (DAPI) (Vector Laboratories, Burlingame, CA, USA). 3,3-Diaminobenzidine (DAB) staining was performed using the ABC HRP kit (Vector Laboratories) and DAB substrate kit (GBI Labs, Bothell, WA, USA), and Nissl staining was performed using cresyl violet solution (Millipore, Burlington, MA, USA) according to the manufacturer’s instructions. Progressive hematoxylin and eosin (H&E) staining was performed as previously described^71^. Supplementary Table 2 presents the primary and secondary antibodies used in this study. The sections of embryonic brain were scanned using an Olympus IX71 microscope equipped with a DP71 digital camera (Olympus, Tokyo, Japan) or Zeiss LSM700 confocal microscope (Carl Zeiss, Oberkochen, Germany). To quantify TH^+^, CC-3^+^, and IBA1^+^ cells, each section was tile-scanned at 20× magnification (4 × 11 tiles) using a Zeiss AxioImager M2 microscope equipped with an AxioCam HRm digital camera to obtain images containing the whole VM. Representative images were obtained using a Zeiss LSM710 confocal microscope (Carl Zeiss). TH-DAB, Nissl, and H&E stained slices were scanned using an Aperio AT2 (Leica Biosystems, Wetzlar, Germany) at 20–40× magnification.

### Quantification of TH^+^ and CC-3^+^ neurons

Unbiased stereological counting of TH^+^ neurons was performed using the ImageJ software (v1.53c, NIH, Bethesda, MD, USA), as described elsewhere^72,73^. Briefly, TH^+^ neurons were counted in every first section (1:10 series; 16 μm per section; each 160 μm apart) at 7–9 coronal levels (beginning from −2.74 to −3.64 mm, relative to the bregma), when co-localized with DAPI (for nuclear staining). The SNc and VTA were delineated according to anatomical landmarks from the Franklin and Paxinos mouse atlas^74^. The counting parameters were as follows: grid size, 100 × 100 μm; counting frame, 50 × 50 μm; height of optical dissector, 13 μm. The calculated CE for each subject was lower than 0.08. To quantify the CC-3^+^ cells among the TH^+^ neurons in the SNc, TH^+^, and CC-3^+^ neurons were counted in five matching coronal slices of the midbrain (from −2.92 to −3.64 mm, relative to the bregma) per mouse.

### Quantification of TH fiber intensity, axonal swelling, and Lewy-like aggregates

To determine the striatal TH fiber intensity, the mean optical density (OD) was measured in TH^+^-stained sections. Images were transformed to gray-scale images of 16 bits, and the background intensity was retracted with the corpus callosum. The OD measurements were determined in three matching coronal slices (1.18, 0.38, and −0.46 mm, relative to the bregma) in the dorsal and ventral parts of the striatum. TH intensity in *Tpbg* KO mice were expressed as a percentage, relative to the intensity in age-matched WT control mice. For quantification of axonal swelling, TH^+^ axonal spheroids were manually counted in the 36 fields (approximately 0.2 mm^2^ area) for the three matching coronal slices (1.18, 0.38, and −0.46 mm, relative to the bregma) per mouse^32,64^. Final counts were calculated and presented as the number of axonal spheroids per 1 mm^2^ area. For quantification of Lewy-like aggregates in the H&E-stained sections, filamentous or spherical eosinophilic structures were manually counted in the 50 fields (approximately 0.4 mm^2^ area) for the five matching coronal slices (from −2.92 to −3.64 mm, relative to the bregma) per mouse. Final counts were calculated and presented as the number of Lewy-like aggregates per 1 mm^2^ area.

### Mouse brain striatum and SNc dissection

For dissection of the mouse brain, the mice were anesthetized with isoflurane, and the whole brain was rapidly removed from the calvarium. The brain was placed on a mouse brain matrix (Zivic, Pittsburgh, PA, USA); 4 mm thick slices of the forebrain and 1 mm thick slices of the midbrain were obtained for the dissection of the striatum and SNc, respectively, under cold and sterile RNase-free conditions. Using anatomical markers^74^, the striatum and SNc were rapidly dissected out from these slices under a stereomicroscope, frozen in dry ice, and stored at −80 °C until analysis.

### Dopamine measurement

For the analysis of dopamine content in the striatum, dissected striatal tissues were homogenized in 0.01 N HCl containing 1 mM EDTA and 4 mM sodium metabisulfite and centrifuged at 13,000 rpm for 20 min. The concentration of dopamine in the supernatant was determined using a dopamine ELISA Kit (Abnova, Walnut, CA, USA), according to the manufacturer’s instructions. Briefly, dopamine in the dialysate was extracted using a cis-diol-specific gel, acylated, and derivatized enzymatically. The optical density was determined at 450 nm using a microplate reader (Bio-Rad, Hercules, CA, USA). Dopamine levels were normalized to the weight of the wet tissue.

### Western blot

The dissected SNc tissues from the right hemisphere were homogenized in RIPA buffer (Thermo Scientific) with 1× proteinase inhibitor and phosphatase inhibitor (Thermo Scientific) and centrifuged at 16,000 rpm at 4 °C for 30 min. The protein concentration of the supernatants was determined using a BCA assay (Thermo Scientific). The proteins (10–15 µg) were denatured at 95 °C for 5 min, separated by SDS-PAGE on a 4–20% polyacrylamide bis-tris gel (iNtRON Biotechnology, Seongnam, South Korea), and transferred to a PVDF membrane (Millipore). The membranes were blocked in 5% skim milk in TBS with 0.1% Tween 20 (TBS-T) and incubated overnight at 4 °C with primary antibodies in blocking solution. The membranes were washed with TBS-T and incubated for 1 h at 25 °C with horseradish peroxidase (HRP)-conjugated secondary antibodies (Supplementary Table 2). The blots were visualized using an ECL Kit (Thermo Scientific), and chemiluminescence images were obtained using a LAS-4000 lumino-image analyzer system (Fujifilm, Tokyo, Japan). The intensity of each band was quantified using ImageJ (v1.53c, NIH) and normalized to β-actin levels.

### Morphological characterization of IBA1-positive microglia

The morphology of IBA1^+^ cells was analyzed and scored in five matching coronal slices of the midbrain (from −2.92 to −3.64 mm, relative to the bregma) per mouse, according to a previous method^33,34^. On the basis of their morphological characteristics, IBA1^+^ cells were classified as resting microglia (type A, visible thin cytoplasm with long and thin processes), activated microglia (type B, dense and enlarged cell body with thick, short processes), and phagocytic microglia (type C, pseudo-amoeboid shape, large, dark cell body merging with processes).

### Behavioral assessments

For behavioral tests, WT, *Tpbg* Hem, and *Tpbg* KO mice were obtained through intercross breeding of *Tpbg* Hem mice. Adult female mice at a young age (WT mice, *n* = 9; *Tpbg* KO mice, *n* = 5) and old age (WT mice, *n* = 9; *Tpbg* Hem mice, *n* = 6; *Tpbg* KO mice, *n* = 9) were examined. All behavioral experiments were performed during the light phase of the light/dark cycle, and the mice were habituated to the testing room for 1 h before the tests. All behavioral tests were conducted and analyzed in a genotype-blinded manner. For gait analysis^75,76^, a 45 cm runway was placed between a bright light source and the home cage in a darkened room. The mice were trained over two consecutive days to walk across a 45 cm runway, which led to their home cage. On the day of testing, the fore and hind paws were painted with non-toxic ink of different colors, and they were allowed to walk on absorbent paper placed on the runway. The ink footprints were analyzed to measure stride length, stride width, and intra-step distance. For the challenging beam travel test^77^, an acrylic beam was used consisting of four segments (25 cm each, 1 m total length) of varying widths (3.5, 2.5, 1.5, and 0.5 cm) and a wire mesh (1 cm width) of the corresponding width on each beam surface. The mice were trained to traverse the entire length of the beam without the mesh grid to their home cage for two consecutive days. On the day of the test, the mice were videotaped while traversing the grid-surfaced beam for a total of five trials. The time to traverse, number of steps, and number of errors were counted by viewing the videotapes in slow motion. For the adhesive removal test^77^, a small adhesive stimulus was placed on the snout of the mouse using a tweezer. The mouse was placed back in its home cage and timed until it made contact with the stimulus (time to contact) and removed (time to removal) the stimulus. Each mouse underwent three trials with a cutoff of 1 min and an inter-trial interval of 2 min.

### Analysis of TPBG-interacting proteins

To investigate the impact of alteration of *Tpbg* expression on the pathophysiology of PD, we identified TPBG-interacting proteins from the STRING database with a cutoff value of 0.4 (moderate confidence)^35^; the study yielded 36 proteins. Cytoscape^78^ and MCODE^36^ were used to identify dense clusters in the network of TPBG-interacting proteins. For functional analysis, enrichment analysis using DAVID^37^ was performed.

### Acquisition and analysis of RNA-seq data

For RNA-seq analysis, total RNA was extracted from the dissected SNc tissues (Supplementary Fig. 11a) (old age; WT mice *n* = 2; *Tpbg* KO mice, *n* = 2) using the Easy-Spin^®^ Total RNA Extraction kit (iNtRON Biotechnology) according to the manufacturer’s instructions. Total RNA from each sample was processed and analyzed by Macrogen Inc. (Seoul, South Korea) for paired-end sequencing, with the aim of generating over 60 million reads. Adapter sequences were trimmed using BBduk, and transcript quantification was performed using Salmon^79^, using the reference transcript GENCODEvM23. To identify differentially expressed genes, transcript abundance estimates were imported into DESeq2^80^ using tximport^81^ for further analysis. A correlation plot was generated using ggplot2 (v3.3.3), and a heatmap was generated using pheatmap (v1.0.12). GSEA preranked analysis was performed using fgsea (v1.16.0)^82^.

### Statistical analysis

Statistical analyses were performed using GraphPad Prism v9.0.2. (GraphPad Software, San Diego, CA, USA). For neurohistological and neurochemical analysis, data are presented as the mean ± standard deviation (SD) or standard error of the mean (SEM) of at least three individual subjects. Statistical significance was determined using the Mann–Whitney test for the comparison of genotypes (WT vs. *Tpbg* KO) or two-way ANOVA with Sidak’s multiple comparison test for the comparison of ages (young vs. old) and genotypes (WT vs. *Tpbg* KO). For behavior measurements, data are presented as the mean ± SEM. Differences between ages (young vs. old) and genotypes (WT vs. *Tpbg* KO) were analyzed using two-way ANOVA with Sidak’s multiple comparison test. Differences between WT, *Tpbg* Hem, and *Tpbg* KO mice at old age were analyzed using the Kruskal–Wallis test with Dunn’s multiple comparison test. Significant differences were described in the graph when the *p*-value was less than 0.05 and assumed at *p < 0.05, **p < 0.01, ***p < 0.001, and ****p < 0.001.

### Reporting summary

Further information on research design is available in the Nature Research Reporting Summary linked to this article.

## Supplementary information

Supplementary Information

Reporting Summary

## Data Availability

RNA-seq data have been deposited in the Gene Expression Omnibus (GEO) database under accession codes “GSE178400”. All other relevant data supporting the key findings of this study are available within the article and its Supplementary Information file.
