# Supplementary material for: Trophoblast glycoprotein is a new candidate gene for Parkinson’s disease
Source: NPJ Parkinsons Dis. 2021 Dec 7;7:110. doi: 10.1038/s41531-021-00252-0 (PMC8651753; doi:10.1038/s41531-021-00252-0)
Supplement: Supplementary file 1 — Supplementary Information [file 41531_2021_252_MOESM1_ESM.pdf]

Supplementary Information

Trophoblast glycoprotein is a new candidate gene for Parkinson's disease

Sanghyun Park, Jeong-Eun Yoo, Gyu-Bum Yeon, Jin Hee Kim, Jae Souk Lee, Sung Kyoung Choi, Young-Gi Hwang, Chan Wook Park, Myung Soo Cho, Jongwan Kim, Dokyun Na, Hyung Wook Kim, Dae-Sung Kim*, and Dong-Wook Kim*

* Correspondence should be addressed to D.-S.K. (sonnet10@korea.ac.kr) or D.-W.K. (dwkim2@yuhs.ac)

Supplementary Figure 1

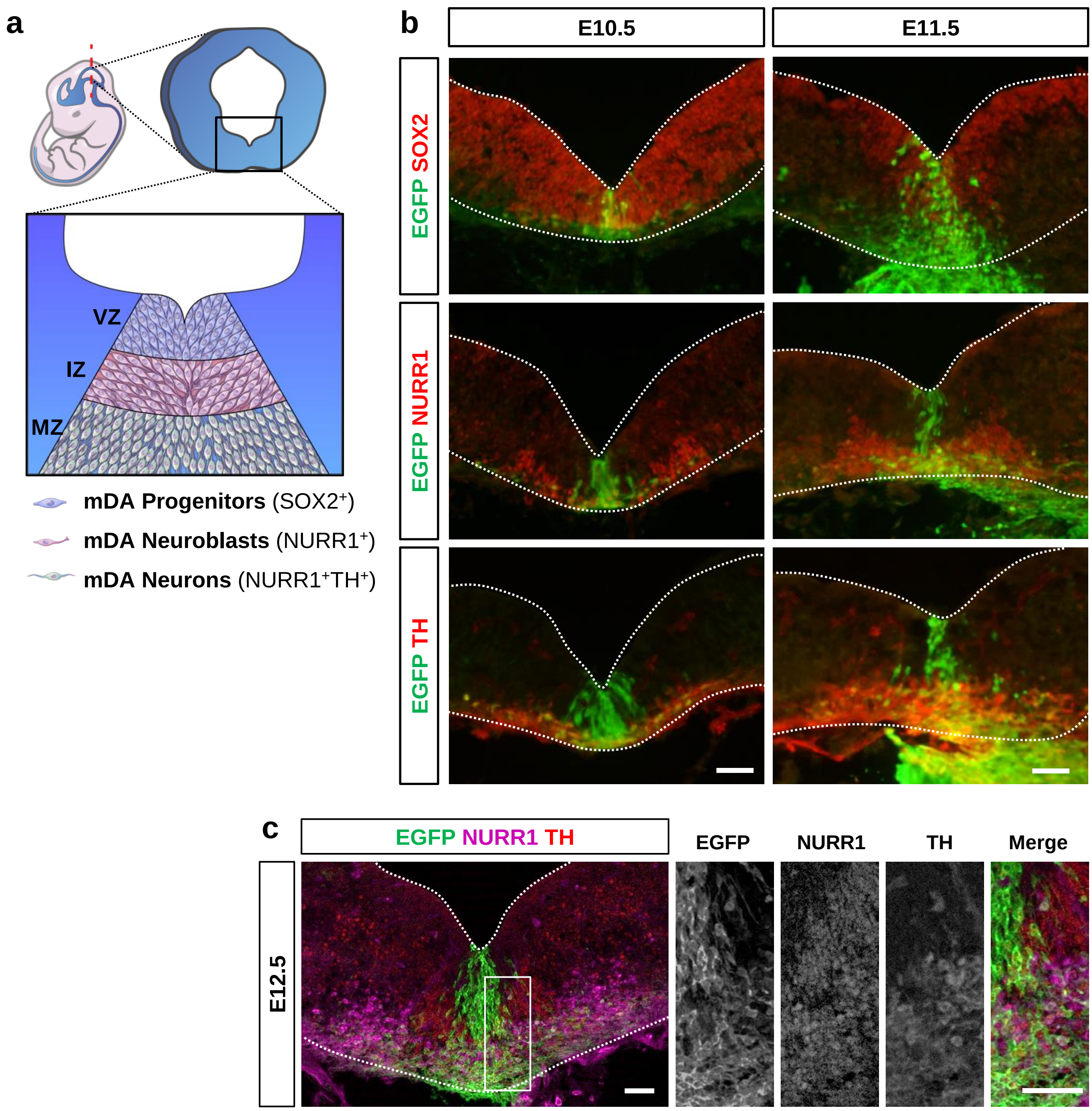

Supplementary Figure 1. Analysis of spatiotemporal expression of TPBG during mDA development. a Schematic representation of the floor plate of the ventral midbrain during mDA development. **b-c** Between E10.5 and E12.5, in the coronal section of the mouse embryo brain, EGFP-expressing cells co-express mDA stage-specific markers (SOX2, NURR1, and TH) according to their location (VZ, ventricular zone; IZ, intermediate zone; and MZ, marginal zone) (Scale bar: 50 μm).

Supplementary Figure 2

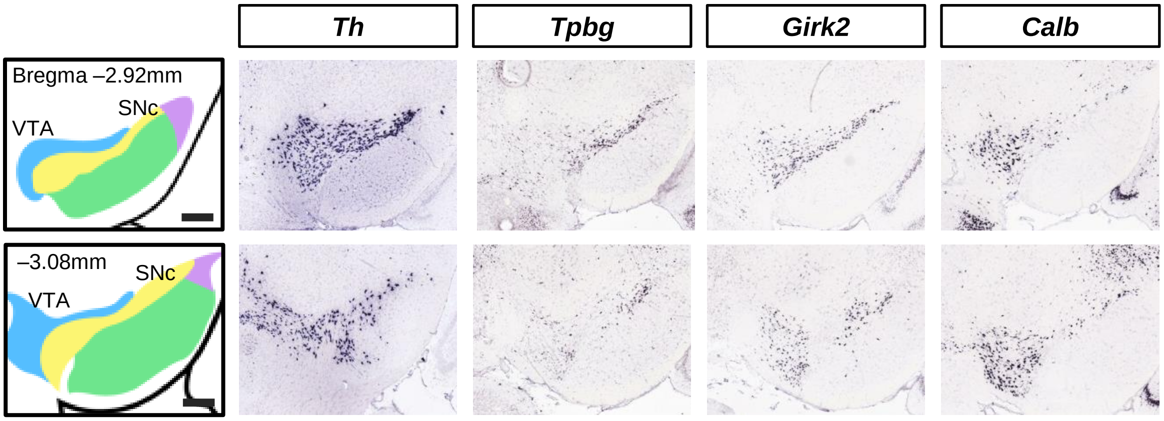

Supplementary Figure 2. *In situ* hybridization for *Tpbg* and mDA markers in the postnatal mouse brain from Allen institute. *In situ* hybridization at postnatal day 56 showing *Tpbg* mRNA expressed in mDA subsets, characterized by the expression of *Th*, *Girk2*, and *Calbindin1* (*Calb*) (data from Allen Institute for Brain Science) (Scale bar: 250 μm).

Supplementary Figure 3

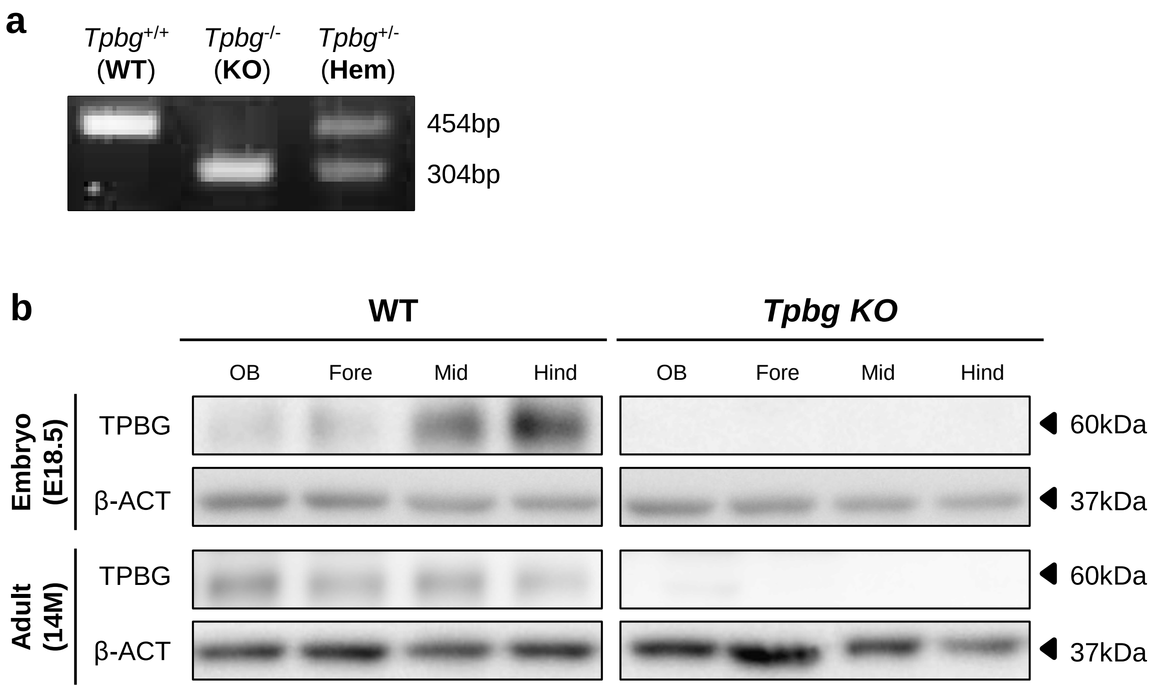

Supplementary Figure 3. Confirmation of *Tpbg* knockout. a Genotyping was performed using a PCR-based strategy utilizing appropriate primer sets that yielded a 454 bp band in wild-type (WT) mice, a 304 bp band in *Tpbg* knockout (KO) mice, and both bands in *Tpbg* hemizygous (Hem) mice. **b** Western blot analysis shows that TPBG expression was detected in the brain of WT mice at the embryo (E18.5) and adult (14 months old, 14M) stages but not in the brain of *Tpbg* KO mice at either stage. A housekeeping protein, β-actin (β-ACT), was used as the positive control. Molecular size markers are shown in kilodaltons (kDa). Un-cropped images of blots are shown in the Supplementary Fig. 12a. OB, olfactory bulb; Fore, forebrain; Mid, midbrain; Hind, hindbrain.

Supplementary Figure 4

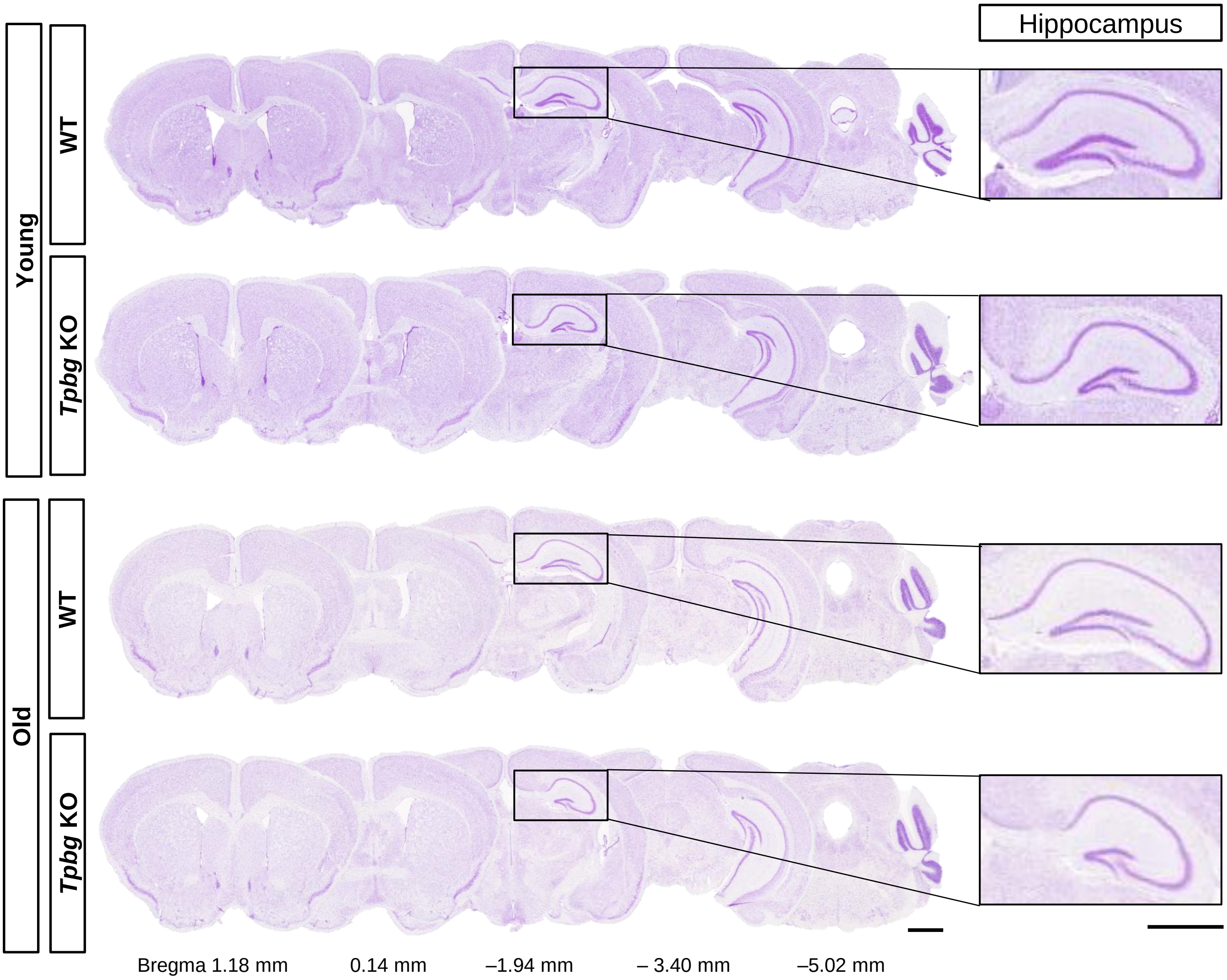

Supplementary Figure 4. Histopathological evaluation of brain structure in the *Tpbg* KO mice. According to representative Nissl-stained images of coronal sectioned brain, the brain of *Tpbg* KO mice exhibit disorganization of the hippocampus but no structural abnormality in other regions. Coronal sections are arranged in order from the rostral to caudal sections (Scale bar: 1 mm).

Supplementary Figure 5

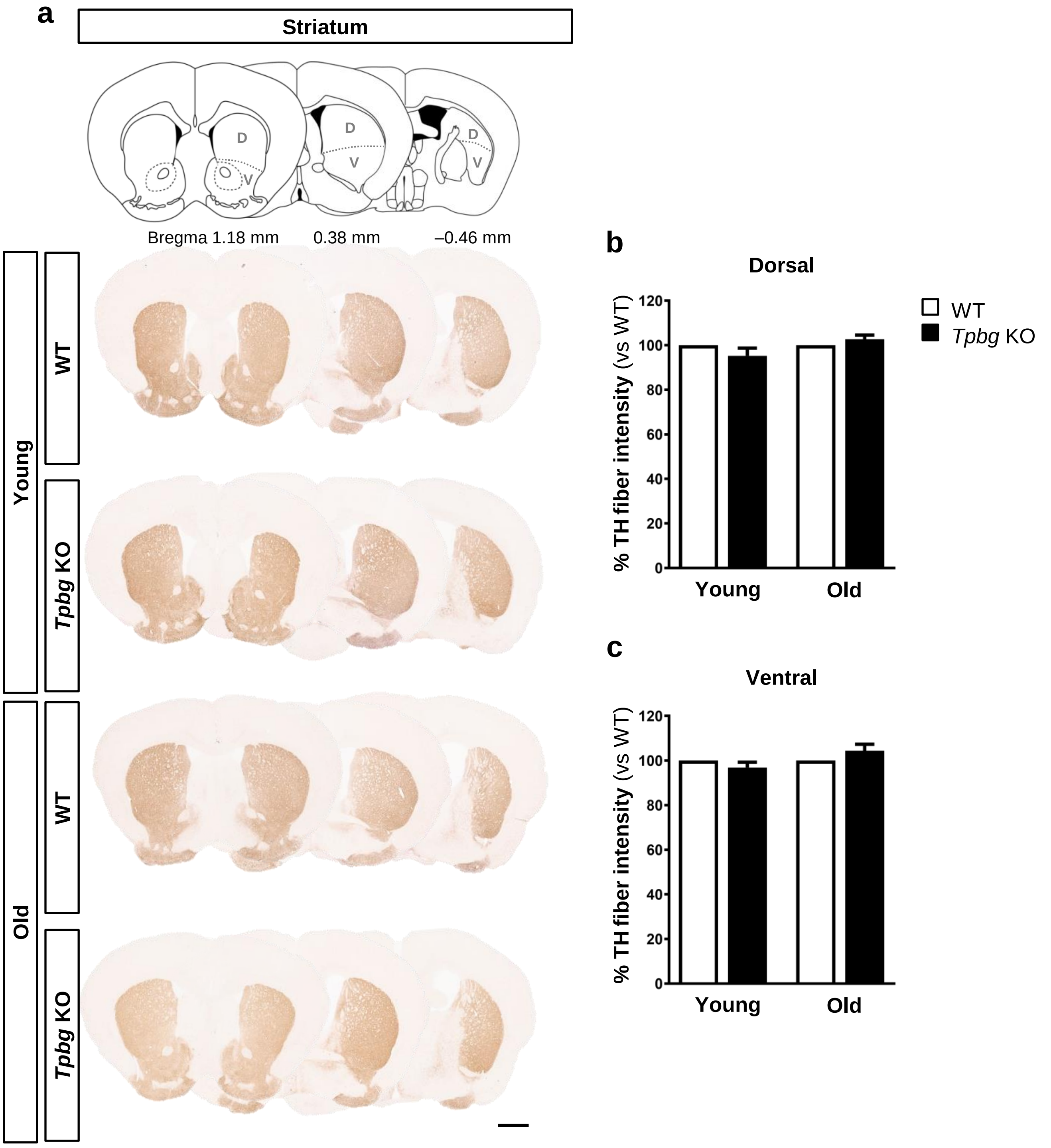

Supplementary Figure 5. Investigation of TH^{+} fiber density in the striatum. a Representative images of DAB staining for TH in the striatum of WT and *Tpbg* KO mice at young and old ages (Scale bar: 1 mm). **b-c** Quantification of optical intensity for TH^{+} fiber in the dorsal (**b**) and ventral (**c**) striatum relative to WT control. Bars represent % striatal TH^{+} fiber intensity in the *Tpbg* KO mice relative to the WT counterpart (WT was set at 100%). Data is represented as the mean ± SD (young; WT, n=3; KO, n=3; old; WT, n=3; KO, n=3) (not significant, $P > 0.05$; Mann–Whitney test).

Supplementary Figure 6

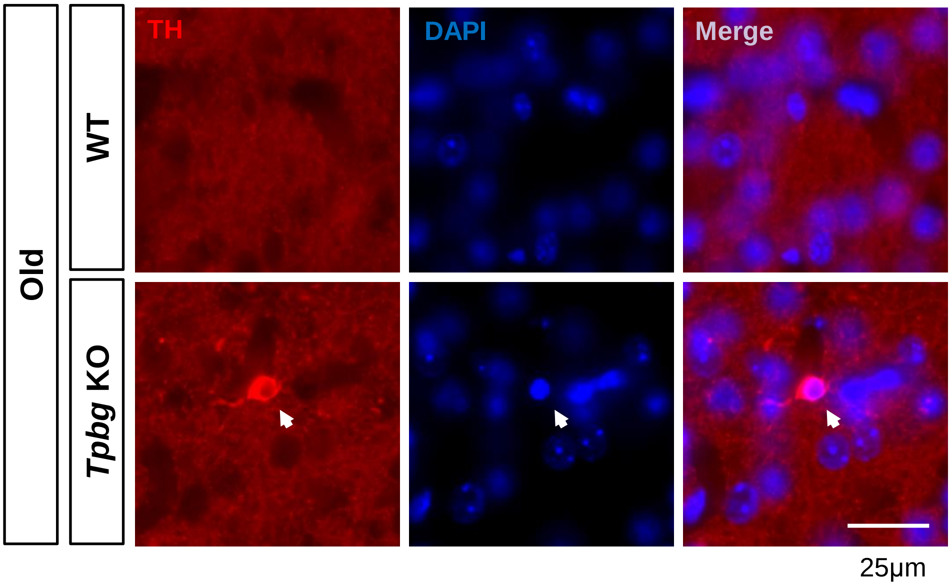

Supplementary Figure 6. Investigation of abnormal mDA nerve terminals in the striatum of the aged *Tpbg* KO mice Representative image of sporadic TH^+ cell bodies (arrow) detected in the striatum of aged *Tpbg* KO mice (Scale bar: 25 μm).

Supplementary Figure 7

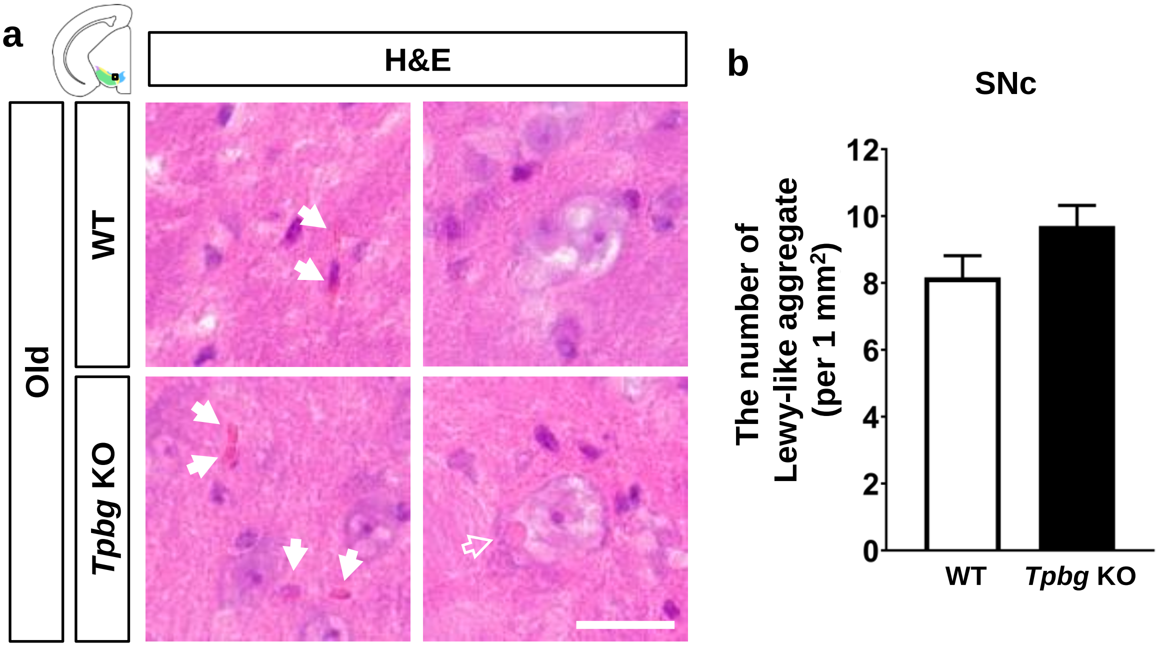

Supplementary Figure 7. Lewy-like aggregates in the SNc. **a** Lewy-like aggregates detected with hematoxylin & eosin (H&E) staining as filamentous (solid arrow) or spherical (open arrow) eosinophilic structures in the SNc of WT and *Tpbg* KO mice at old age. **b** Quantification of the number of Lewy-like aggregates in the SNc of WT (n=3) and *Tpbg* KO (n=3) mice at old age. Data is represented as the mean ± SD (not significant, $P > 0.05$; Mann–Whitney test).

Supplementary Figure 8

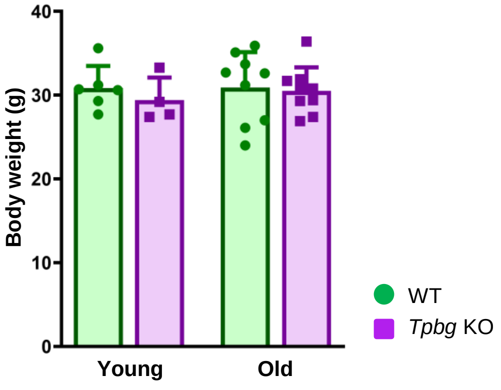

Supplementary Figure 8. Body weight. Body weights of WT and *Tpbg* KO mice measured at young (WT, n=6; KO, n=4) and old age (WT, n=9; KO, n=9) before the behavior test. Data is represented as the mean ± SD (not significant, $P > 0.05$; Two-way ANOVA with Sidak's multiple comparison test).

Supplementary Figure 9

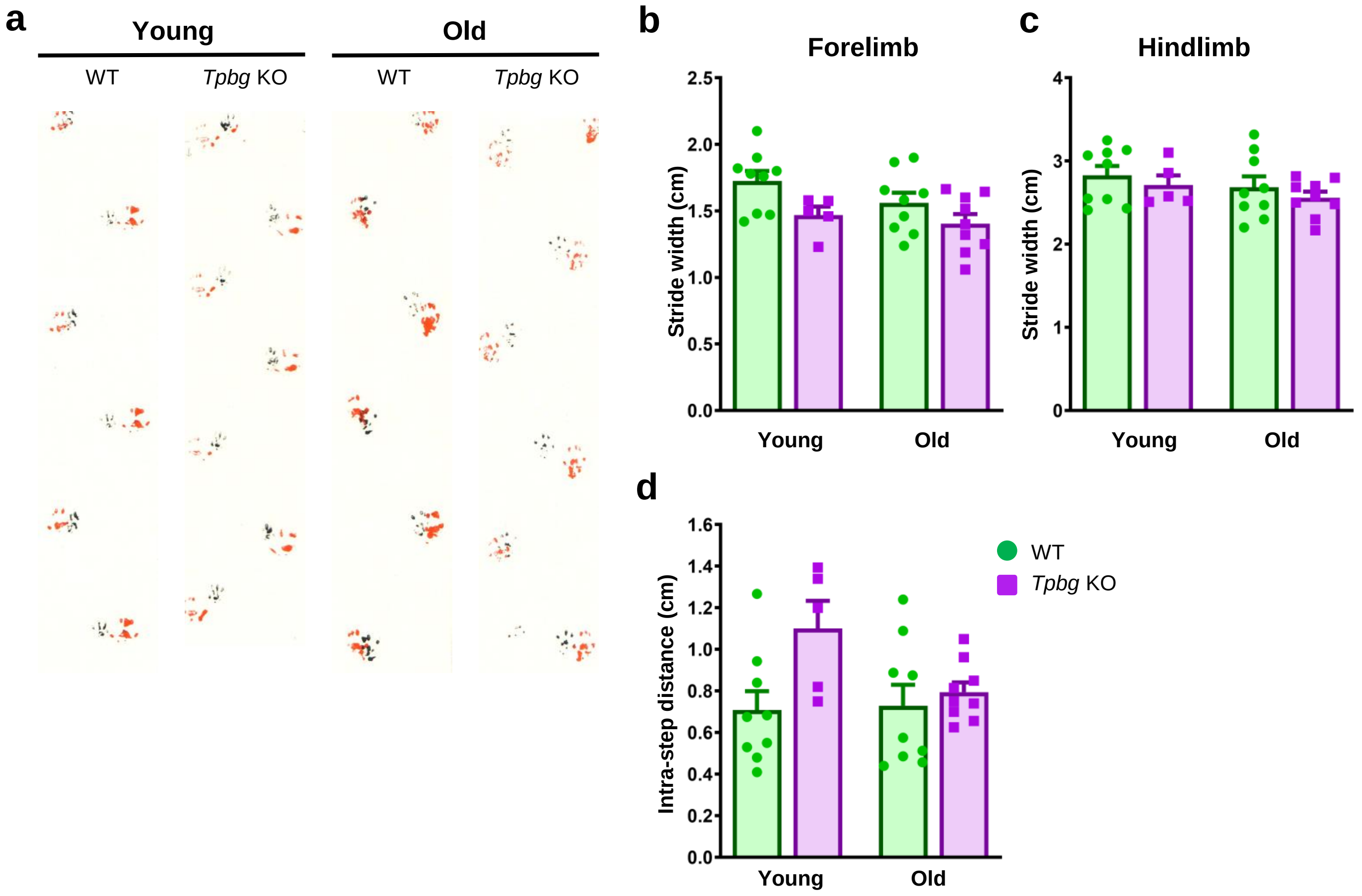

Supplementary Figure 9. Assessment of gait patterns. a Representative images of gait analysis footprint for WT and *Tpbg* KO mice at young (WT, n=9; KO, n=5) and old (WT, n=9; KO, n=9) age. **b–d** Gait pattern measurements of forelimb (**b**) and hindlimb (**c**) stride width and intra-step distance (**d**). Data is represented as the means ± SEM (not significant, $P > 0.05$; Two-way ANOVA with Sidak's multiple comparison test).

Supplementary Figure 10

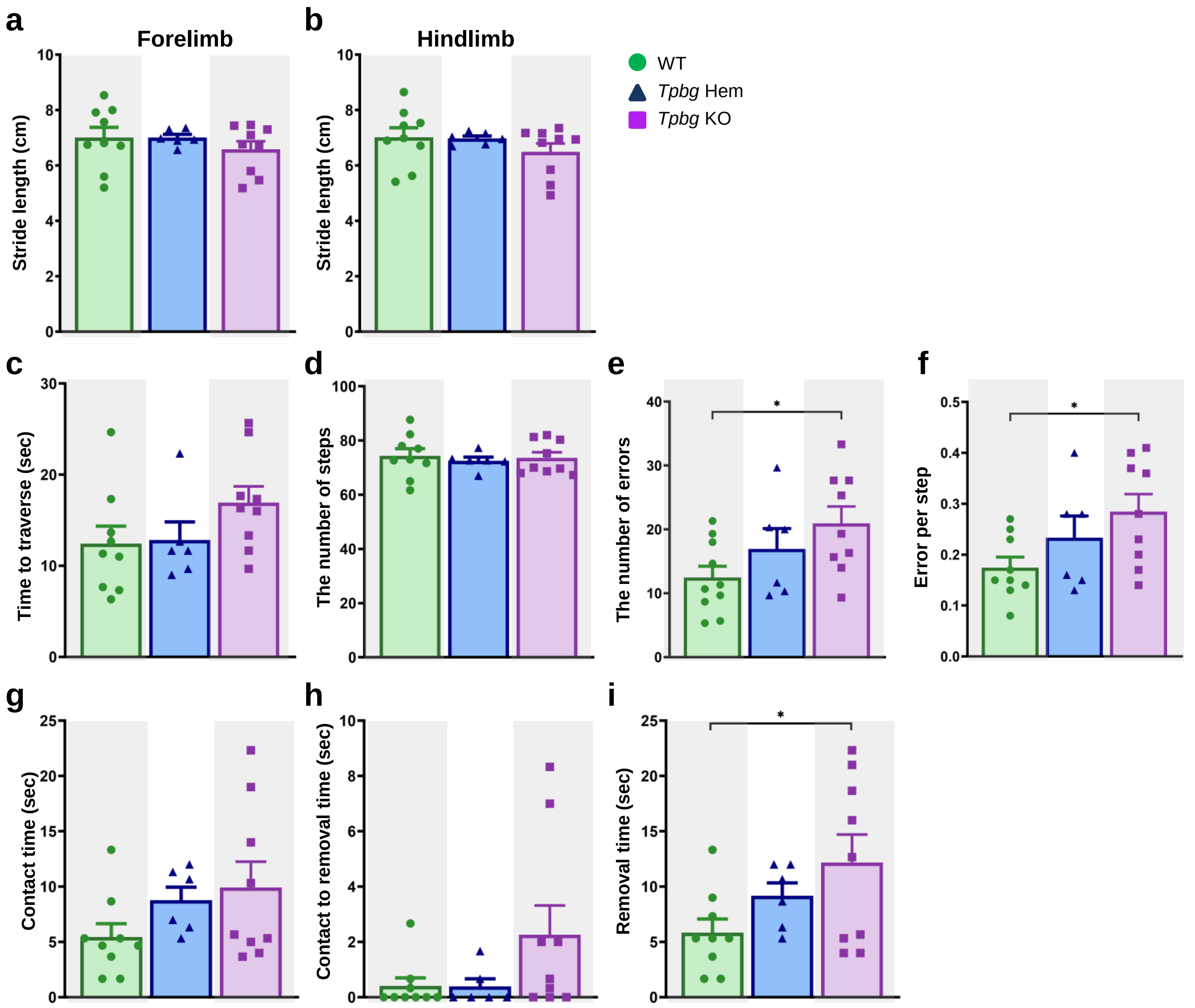

Supplementary Figure 10. Assessment of sensorimotor tests that are sensitive to alterations in the nigrostriatal dopaminergic system. Behavioral examination of aged WT (n=9), *Tpbg* Hem (n=6), and *Tpbg* KO (n=9) mice was performed as follows: **a–b** Gait patterns were measured as forelimb (**a**) and hindlimb (**b**) stride lengths. **c–f** Motor performance and coordination were assessed using the challenging beam travel test, as the time to traverse (**c**), the number of steps (**d**), the number of errors (**e**), and errors per step (**f**). **g–i** Sensorimotor function was assessed by measuring time to contact (**g**), contact to removal time (**h**), and time to removal (**i**) in the adhesive removal test. The values on gray background indicate the data for WT and *Tpbg* KO mice used in Figure 6. All the data is represented as the means ± SEM (* $P < 0.05$; Kruskal–Wallis test with Dunn's multiple comparison test).

Supplementary Figure 11

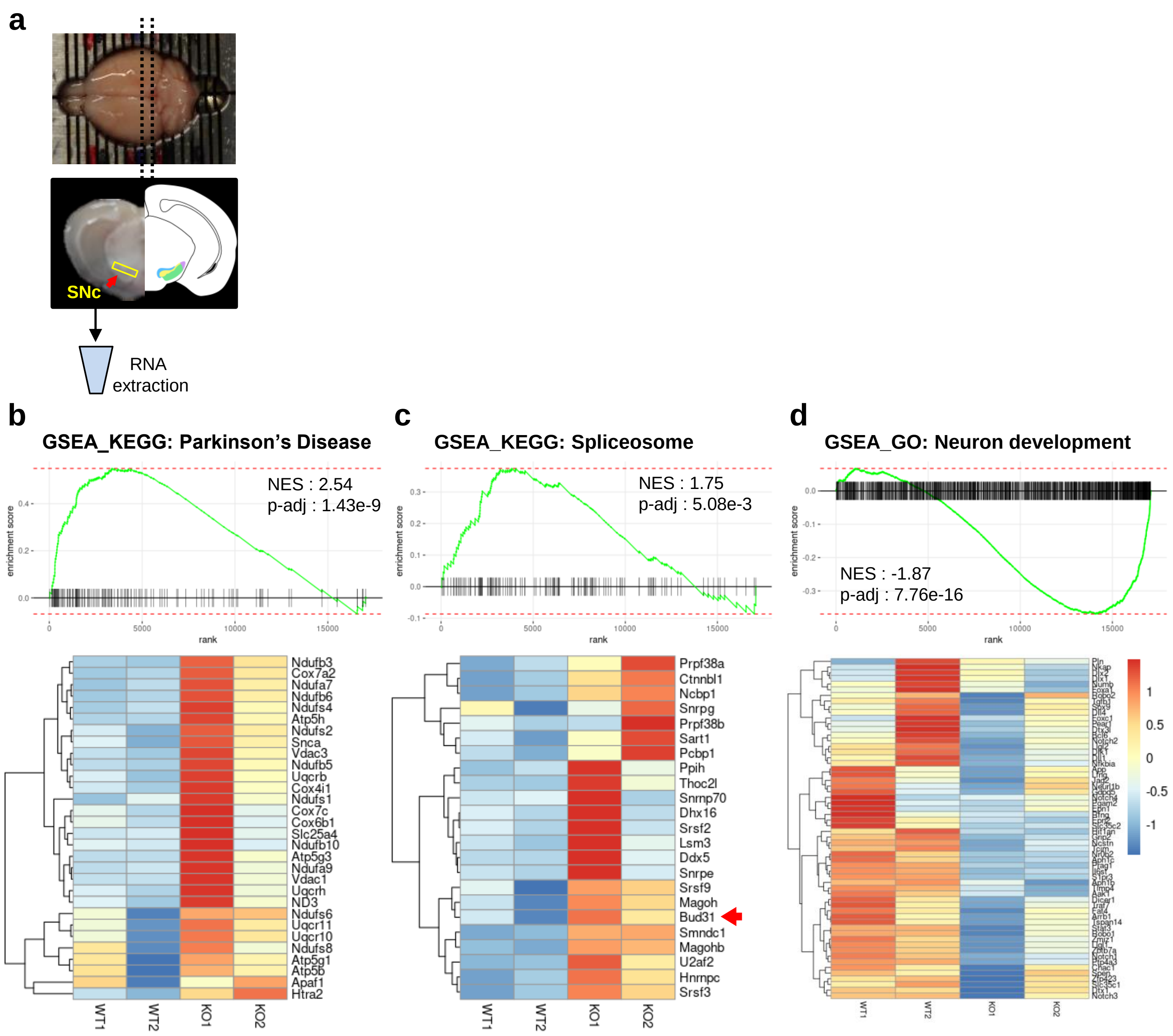

Supplementary Figure 11. Global transcriptome alterations in the SNc of aged *Tpbg* KO mice. a Diagrammatic representation of dissection of the SNc tissue from the mouse brain. For isolating midbrain slices, coronal cuts were made, as indicated by the dotted lines, approximately at the bregma –2.90 mm and –3.90 mm. Subsequently, the SNc tissue was dissected out from the midbrain slice, as indicated by the yellow solid line. **b-d** Gene set enrichment analysis (GSEA) preranked analysis of RNA-seq data comparing the *Tpbg* KO SNc vs. WT SNc using the KEGG pathway and GeneOntology (GO) database. The upper panels are GSEA enrichment plots of selected KEGG pathway – Parkinson's Disease (**b**) and Spliceosome (**c**) and GO – Neuron development (**d**); the bottom panels are heatmaps of each leading edge genes. NES, normalized enrichment score.

Supplementary Figure 12

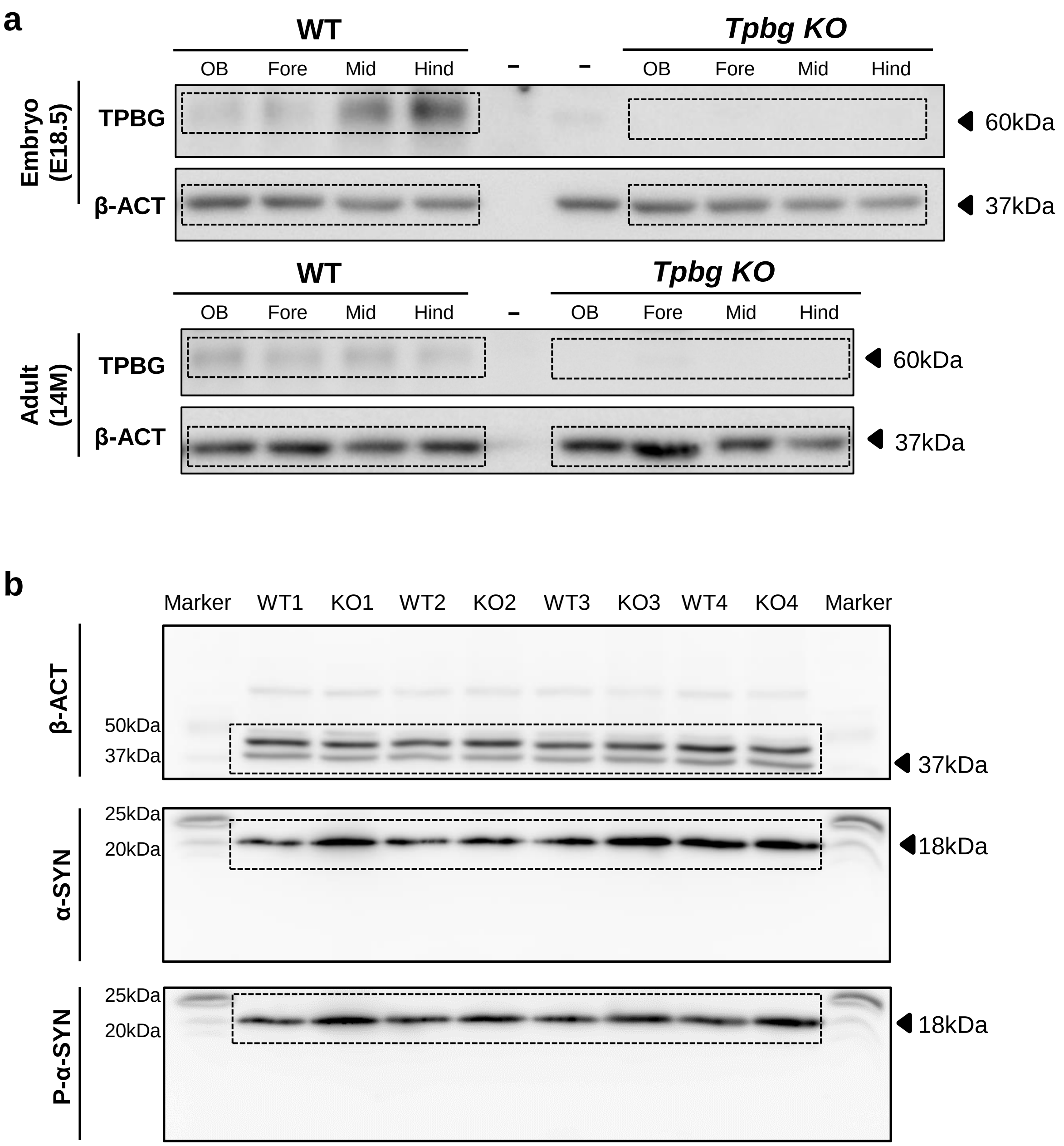

Supplementary Figure 12. Uncropped blots of Supplementary figure 3 and figure 4 with molecular weight markers. a. Uncropped blots of supplementary figure 3b; detection of TPBG and β-ACT in the WT and *Tpbg* KO mice brain at the embryo (E18.5) and adult (14 months old, 14M) stages. The parts cut along a dotted line were used as supplementary figure 3b. **b.** Uncropped blots of figure 4a; detection of β-ACT, α-SYN, and P-α-SYN in the SNc of WT and *Tpbg* KO mice at old age. The parts cut along a dotted line were used as figure 4a. Samples derive from the same experiment and gels/blots were processed in parallel.

Supplementary Table 1. PCR primer sequences for genotyping

Primer		Sequence (5' to 3')	Length (mer)	Prodcut Size (bp)
Tpbg-EGFP line	Actin control -forward	GAT GAC GAT ATC GCT GCG CTG GTC G	25	1000 (WT&TG)
	Actin control -reverse	GCC TGT GGT ACG ACC AGA GGC ATA CAG	27	
	EGFP-forward	CCT ACG GCG TGC AGT GCT TCA GC	23	300 (TG)
	EGFP-reverse	CGG CGA GCT GCA CGC TGC GTC CTC	24	
Tpbg KO line	Neo3A	GCA GCG CAT CGC CTT CTA TC	20	454 (WT)<br>304 (KO)
	DNA321-20	ACA GAA CAC ACC GGG ATT	18	
	DNA321-19	AGG CTT ACC TGC GCA TTC	18	

(WT, wild type; TG, transgenic; KO, knockout)

Supplementary Table 2. List of antibodies used in this study

Antibody	Target Protein	Company	Species
Primary Antibody	EGFP	Rockland	Goat
	EGFP	Rockland	Mouse
	TPBG	R&D System	Sheep
	LMX1A	Santa Cruz	Goat
	FOXA2	Abcam	Rabbit
	NURR1	Santa Cruz	Rabbit
	TH	Pel-Freez	Rabbit
	TH	Pel-Freez	Sheep
	TH	Sigma-Aldrich	Mouse
	SOX2	Millipore	Rabbit
	GIRK2	Almone Labs	Rabbit
	CALBINDIN (CALB)	Millipore	Rabbit
	cleaved CASPASE-3 (CC-3)	Cell Signaling	Rabbit
	α-SYNUCLEIN (α–SYN)	Abcam	Rabbit
	phospho S129-α-SYNUCLEIN (P-α–SYN)	Cell Signaling	Rabbit
	IBA1	Wako	Rabbit
Secondary Antibody	Rabbit IgG (H&L), Alexa Fluor® 594	Thermo Scientific	Donkey
	Rabbit IgG (H&L), Alexa Fluor® 488	Thermo Scientific	Donkey
	Sheep IgG (H&L), Alexa Fluor® 594	Thermo Scientific	Donkey
	Sheep IgG (H&L), Alexa Fluor® 647	Thermo Scientific	Donkey
	Goat IgG (H&L), Alexa Fluor® 488	Thermo Scientific	Donkey
	Goat IgG (H&L), Alexa Fluor® 647	Thermo Scientific	Donkey
	Mouse IgG (H&L), Alexa Fluor® 488	Thermo Scientific	Donkey
	Rabbit IgG (H&L), horseradish peroxidase(HRP)	Invitrogen	Goat
	Sheep IgG (H&L), horseradish peroxidase(HRP)	Invitrogen	Donkey
