# Supplementary material for: Trophoblast glycoprotein is a new candidate gene for Parkinson’s disease
Source: NPJ Parkinsons Dis. 2021 Dec 7;7:110. doi: 10.1038/s41531-021-00252-0 (PMC8651753; doi:10.1038/s41531-021-00252-0)
Supplement: Supplementary file 2 — Reporting Summary [file 41531_2021_252_MOESM2_ESM.pdf]

nature portfolio

Corresponding author(s): Dong-Wook Kim, Dae-Sung Kim

Last updated by author(s): Jun 24, 2021

Reporting Summary

Nature Portfolio wishes to improve the reproducibility of the work that we publish. This form provides structure for consistency and transparency in reporting. For further information on Nature Portfolio policies, see our Editorial Policies and the Editorial Policy Checklist.

Statistics

For all statistical analyses, confirm that the following items are present in the figure legend, table legend, main text, or Methods section.

n/a	Confirmed	
☐	☒	The exact sample size (*n*) for each experimental group/condition, given as a discrete number and unit of measurement
☐	☒	A statement on whether measurements were taken from distinct samples or whether the same sample was measured repeatedly
☐	☒	The statistical test(s) used AND whether they are one- or two-sided<br>*Only common tests should be described solely by name; describe more complex techniques in the Methods section.*
☐	☒	A description of all covariates tested
☐	☒	A description of any assumptions or corrections, such as tests of normality and adjustment for multiple comparisons
☐	☒	A full description of the statistical parameters including central tendency (e.g. means) or other basic estimates (e.g. regression coefficient) AND variation (e.g. standard deviation) or associated estimates of uncertainty (e.g. confidence intervals)
☐	☒	For null hypothesis testing, the test statistic (e.g. *F*, *t*, *r*) with confidence intervals, effect sizes, degrees of freedom and *P* value noted<br>*Give P values as exact values whenever suitable.*
☒	☐	For Bayesian analysis, information on the choice of priors and Markov chain Monte Carlo settings
☒	☐	For hierarchical and complex designs, identification of the appropriate level for tests and full reporting of outcomes
☒	☐	Estimates of effect sizes (e.g. Cohen's *d*, Pearson's *r*), indicating how they were calculated

Our web collection on statistics for biologists contains articles on many of the points above.

Software and code

Policy information about availability of computer code

Data collection	Zeiss AxioImager M2 microscope, Zeiss LSM710 confocal microscope, Aperio AT2 slide scanner, and Illumina NovaSeq 6000
Data analysis	ZEN 3.1, ImageScope software v12.3.0.506, Fiji-ImageJ v1.53c, GraphPad Prism v9.0.2., and Illumina Casava1.8 Software

For manuscripts utilizing custom algorithms or software that are central to the research but not yet described in published literature, software must be made available to editors and reviewers. We strongly encourage code deposition in a community repository (e.g. GitHub). See the Nature Portfolio guidelines for submitting code & software for further information.

Data

Policy information about availability of data

All manuscripts must include a data availability statement. This statement should provide the following information, where applicable:

- Accession codes, unique identifiers, or web links for publicly available datasets
- A description of any restrictions on data availability
- For clinical datasets or third party data, please ensure that the statement adheres to our policy

RNA-seq data have been deposited in the Gene Expression Omnibus (GEO) database under accession codes “GSE178400". All other relevant data supporting the key findings of this study are available within the article and its Supplementary Information file.

Field-specific reporting

Please select the one below that is the best fit for your research. If you are not sure, read the appropriate sections before making your selection.

☒ Life sciences ☐ Behavioural & social sciences ☐ Ecological, evolutionary & environmental sciences

For a reference copy of the document with all sections, see nature.com/documents/nr-reporting-summary-flat.pdf

Life sciences study design

All studies must disclose on these points even when the disclosure is negative.

Sample size	No statistical analysis was performed to predetermine sample size, but 3-9 mice per group were used for each analysis, which are standard numbers for the field.
Data exclusions	No data were excluded
Replication	All attempts at replication were successful.
Randomization	Randomization was not relevant to this study. Female mice were divided in groups based on their age (young; old) and genotype (WT; Tpbg Hem; Tpbg KO) groups.
Blinding	Immunohistochemical and behavioral analyses were conducted and analyzed by experiments blinded to the groups.

Reporting for specific materials, systems and methods

We require information from authors about some types of materials, experimental systems and methods used in many studies. Here, indicate whether each material, system or method listed is relevant to your study. If you are not sure if a list item applies to your research, read the appropriate section before selecting a response.

Materials & experimental systems

n/a	Involved in the study	
☐	☒	Antibodies
☒	☐	Eukaryotic cell lines
☒	☐	Palaeontology and archaeology
☐	☒	Animals and other organisms
☒	☐	Human research participants
☒	☐	Clinical data
☒	☐	Dual use research of concern

Methods

n/a	Involved in the study	
☒	☐	ChIP-seq
☒	☐	Flow cytometry
☒	☐	MRI-based neuroimaging

Antibodies

Antibodies used	We provide the all the essential information for antibodies in Supplementary Table 2.
Validation	All antibodies used are commercially available and were commercially validated. They were also validated by the previous publications or routine experimental protocols.

Animals and other organisms

Policy information about studies involving animals; ARRIVE guidelines recommended for reporting animal research

Laboratory animals	Tpbg-EGFP mice [strain Tg(TPBG-EGFP) NJ116 Gsat] and Tpbg KO mice (strain Tpbgtm1Lex) from MMRRC were backcrossed with C57BL/6N mice from Orient Bio.<br>Tpbg-EGFP mice were used as follows: 9 embryos between E9.5 and E15.5 (E9.5, n=1; E10.5, n=2; E11.5, n=2; E12.5, n=2; E13.5, n=1; E15.5, n=1) and two adult mice (9 months old).<br>Tpbg KO mice were used as follows: 47 adult mice (young age, 3–4 months old; WT, n=9; Tpbg KO, n=5; old age, 12–14 months old; WT, n=14, Tpbg KO, n=13; Tpbg Hem, n=6).
Wild animals	This study does not involve wild animals.
Field-collected samples	This study does not involve samples collected from the field.
Ethics oversight	All experimental procedures for this study were approved by the Institutional Animal Care and Use Committee of the Yonsei University Health System (2017-0244; 2020-0308).

Note that full information on the approval of the study protocol must also be provided in the manuscript.
